# Fermi-Level Engineering of Nitrogen Core-Doped Armchair Graphene Nanoribbons

**DOI:** 10.1021/jacs.3c05755

**Published:** 2023-08-23

**Authors:** Ethan Chi Ho Wen, Peter H. Jacobse, Jingwei Jiang, Ziyi Wang, Steven G. Louie, Michael F. Crommie, Felix R. Fischer

**Affiliations:** † Department of Chemistry, University of California, Berkeley, California 94720, United States; ‡ Department of Physics, University of California, Berkeley, California 94720, United States; § Materials Sciences Division, Lawrence Berkeley National Laboratory, Berkeley, California 94720, United States; ∥ Kavli Energy NanoSciences Institute at the University of California Berkeley and the Lawrence Berkeley National Laboratory, Berkeley, California 94720, United States; ⊥ Bakar Institute of Digital Materials for the Planet, Division of Computing, Data Science, and Society, University of California, Berkeley, California 94720, United States

## Abstract

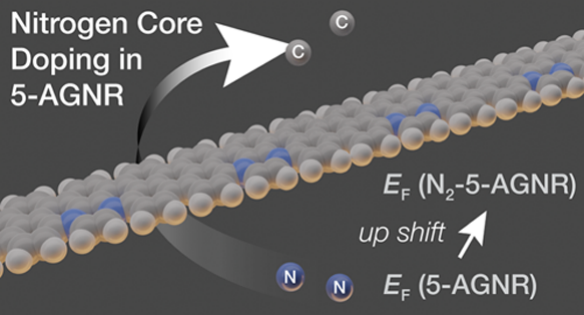

Substitutional heteroatom doping of bottom-up engineered 1D graphene nanoribbons (GNRs) is a versatile tool for realizing low-dimensional functional materials for nanoelectronics and sensing. Previous efforts have largely relied on replacing C–H groups lining the edges of GNRs with trigonal planar N atoms. This type of atomically precise doping, however, only results in a modest realignment of the valence band (VB) and conduction band (CB) energies. Here, we report the design, bottom-up synthesis, and spectroscopic characterization of nitrogen core-doped 5-atom-wide armchair GNRs (N_2_-5-AGNRs) that yield much greater energy-level shifting of the GNR electronic structure. Here, the substitution of C atoms with N atoms along the backbone of the GNR introduces a single surplus π-electron per dopant that populates the electronic states associated with previously unoccupied bands. First-principles DFT-LDA calculations confirm that a sizable shift in Fermi energy (∼1.0 eV) is accompanied by a broad reconfiguration of the band structure, including the opening of a new band gap and the transition from a direct to an indirect semiconducting band gap. Scanning tunneling spectroscopy (STS) lift-off charge transport experiments corroborate the theoretical results and reveal the relationship among substitutional heteroatom doping, Fermi-level shifting, electronic band structure, and topological engineering for this new N-doped GNR.

## Introduction

Graphene nanoribbons (GNRs) represent an emerging class of bottom-up synthesized designer quantum materials whose intrinsic electronic structure can be tuned with atomic precision.^1,2^ GNRs provide access to new physical properties in molecular nanostructures, e.g., antiferromagnetic ordering,^3^ the emergence of symmetry-protected topological phases,^4−7^ and band gap engineering.^8−10^ Switching between a direct and an indirect band gap, however (e.g., by switching the crystal momentum difference between the valence band (VB) maximum and conduction band (CB) minimum), has not been accomplished.^11,12^ Such control over relative band alignments in ***k***-space would have a dramatic impact on the selection rules for optical transitions, exciton lifetimes, and electron–phonon coupling that are relevant for applications in light-harvesting devices,^13,14^ light-emitting diodes,^15−17^ and optoelectronic materials.^18,19^

N-doping GNRs along their edges have been shown to typically result in a downward shift in band energies relative to undoped GNRs.^10,20−30^ Recently, we reported a general approach for embedding a periodic lattice of trigonal planar N atoms into the backbone of bottom-up-synthesized chevron-type GNRs (cGNRs).^31^ The substitution of trigonal planar C atoms by N atoms introduced a single surplus π-electron per N atom that populated emergent dopant states within the semiconducting gap. In this case, charge transfer between the highly localized dopant states in the cGNR and the underlying Au substrate gave rise to a weakly correlated 1D Kondo chain.

**Figure 1:**
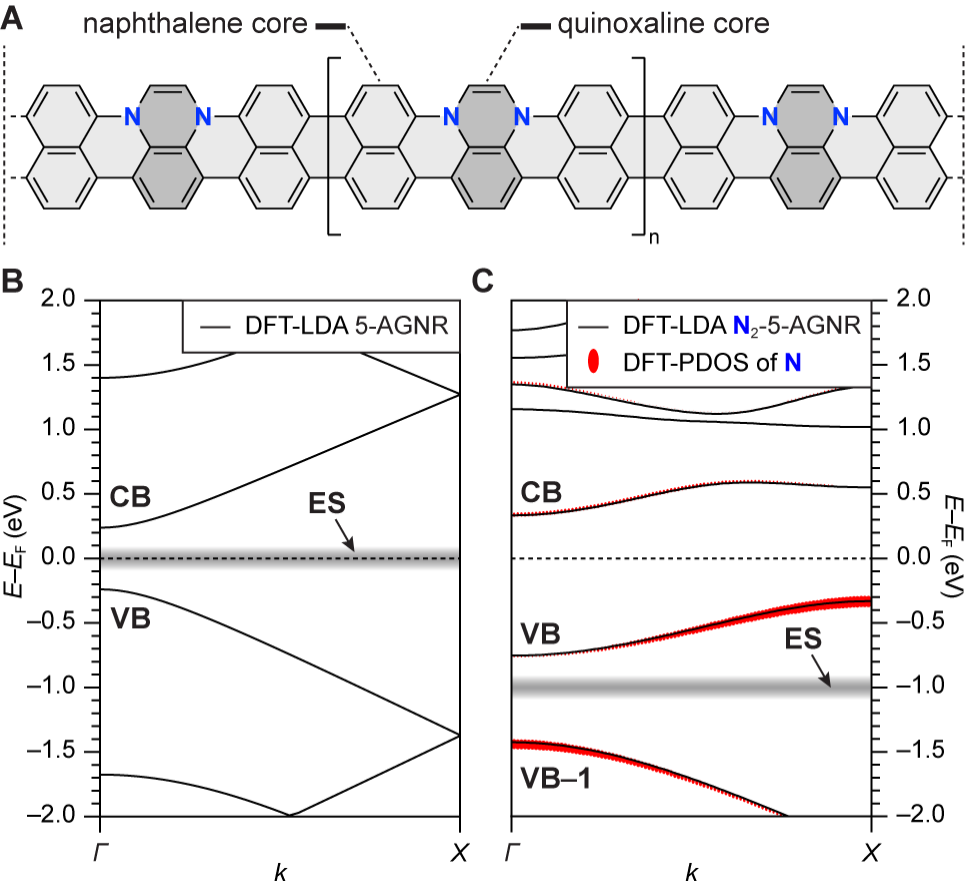
(A) Structure of a N_2_-5-AGNR. (B) DFT-LDA-calculated band structure of all-carbon 5-AGNR. (C) DFT-LDA-calculated band structure of a N_2_-5-AGNR. The relative contribution from orbitals with nitrogen character is shown by the width of the red lines. Features associated with the topological end-state (ES) (obtained from a calculation on a GNR with five repeating units) are indicated by gray lines.

Here, we describe the design and on-surface synthesis of substitutionally nitrogen-doped 5-AGNRs (N_2_-5-AGNRs) derived from a new molecular building block. Figure 1A shows a model of the structure of the new extended N_2_-5-AGNR. The unit cell comprises a quinoxaline core flanked on either side by naphthalene units fused along the short zigzag segments. This unusual orientation places all N atoms along the same armchair edge of the ribbon, effectively breaking mirror symmetry along the main axis of the 5-AGNR. The implications of this new doping pattern on the electronic structure of 5-AGNRs can be seen in *ab initio* density functional theory (DFT) calculations of the band structure. The band structure of an all-carbon 5-AGNR compared to that of a N_2_-5-AGNR is shown in Figure 1B,C. Gray lines at *E* – *E*_F_ = 0.0 and *E* – *E*_F_ = −1.0 eV correspond to the energy of topological end-states (ES) associated with the short zigzag ends of finite 5-AGNR segments for the undoped (0.0 eV) and doped (−1.0 eV) cases.^32^ The width of the red lines in Figure 1C represents the relative contribution of orbitals having nitrogen character to the corresponding bands.

A direct comparison of the Fermi levels of the 5-AGNR band structure versus those of the N_2_-5-AGNR band structure reveals the effect of adding two additional electrons per unit cell due to N atom doping. N_2_-5-AGNR states are seen to be occupied, which can be associated with the highly dispersive CB of the all-carbon 5-AGNR. This band now comprises the new N_2_-5-AGNR VB and has a reduced width of ∼0.4 eV. The new VB is dominated by contributions from orbitals having carbon character at ***k*** = *Γ* (bottom of the VB) and nitrogen character at ***k*** = *X* (top of the VB). The previous degeneracy at ***k*** = *X* is now lifted, and the ***k***-location of the valence band maximum is reversed compared to the 5-AGNR. The *E*_F_ of N_2_-5-AGNRs lies between the top of the VB at ***k*** = *X* and the bottom of the CB at ***k*** = *Γ*, thus producing an indirect gap of ∼0.6 eV. The vertical shift in *E*_F_ (∼1.0 eV) compared to the 5-AGNR is further reflected in the realignment of the topological ES to *E* – *E*_F_ = −1.0 eV.

Besides inducing a direct-to-indirect band gap transformation, the introduction of substitutional N atom dopants brings about a change in the band topology compared to 5-AGNRs. The topological invariant of a GNR with spatial and time reversal symmetry is given by , where *M*_n_(*Γ*) and *M*_n_(*X*) are the mirror symmetry eigenvalues for each filled band *n* at the *Γ* and *X* points, respectively.^4,33^ If one were to evaluate this product over filled bands to the top of VB–1 (VB) in N_2_-5-AGNRs (5-AGNRs), then the  invariant corresponds to a topologically nontrivial nanoribbon (). Because of the new VB for N_2_-5-AGNR, however, the topological invariant of this ribbon becomes . Shifting *E*_F_ via the introduction of nitrogen core dopants in 5-AGNRs is thus also accompanied by a change in the GNR topological order.

## Results and Discussion

### Bottom-up Synthesis of N_2_-5-AGNRs

**Figure 2:**
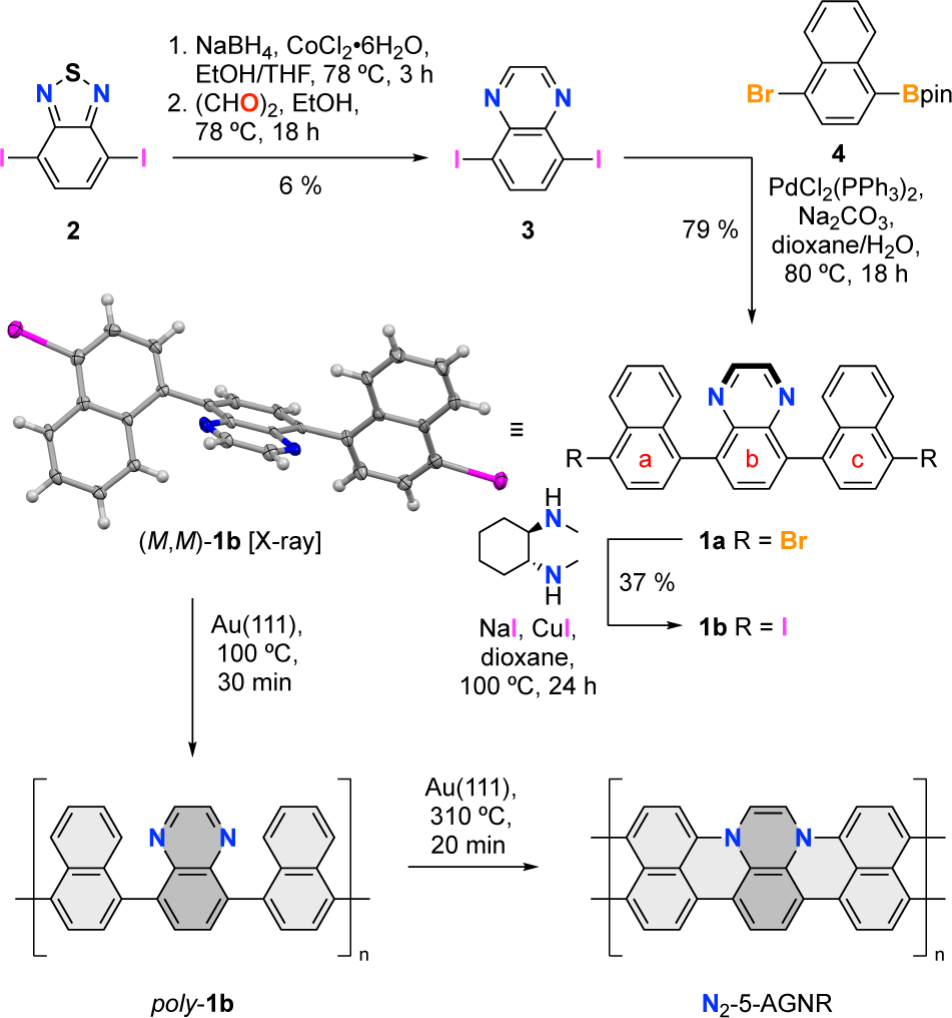
Bottom-up synthesis of N_2_-5-AGNR from molecular precursor **1b**. Single X-ray crystal structure of **1b** is shown. Only the (*M*,*M*) atropisomer is depicted here. Thermal ellipsoids are drawn at the 50% probability level. Color coding: C (gray), I (purple), and N (blue). Hydrogen atoms are placed at calculated positions.

The synthesis of the molecular precursor for N_2_-5-AGNRs, **1b**, is depicted in Figure 2. Reduction of 4,7-diiodobenzo[*c*][1,2,5]thiadiazole (**2**)^34^ followed by condensation with glyoxal yielded 5,8-diiodoquinoxaline (**3**). *Suzuki*–*Miyaura* cross-coupling of **3** with 2 equiv of 2-(4-bromonaphthalen-1-yl)-4,4,5,5-tetramethyl-1,3,2-dioxaborolane (**4**) yielded the parent dinaphthyl quinoxaline **1a**. A subsequent aromatic *Finkelstein* reaction yielded 11,16-diiodo-1,8-diphenyltetrabenzo[*a*,*c*,*h*,*j*]phenazine (**1b**) as the desired monomer precursor. Single crystals suitable for X-ray diffraction and surface-assisted growth were obtained by the slow diffusion of MeCN into a saturated solution of **1b** in CHCl_3_. In the crystal, **1b** adopts quasi-*C*_2_ symmetric axially chiral geometry. The dihedral angles between rings a–b and b–c range from 63.6 to 70.5°. The unit cell contains both atropoenantiomers (*P*,*P*)-**1b** and (*M*,*M*)-**1b**.

**Figure 3:**
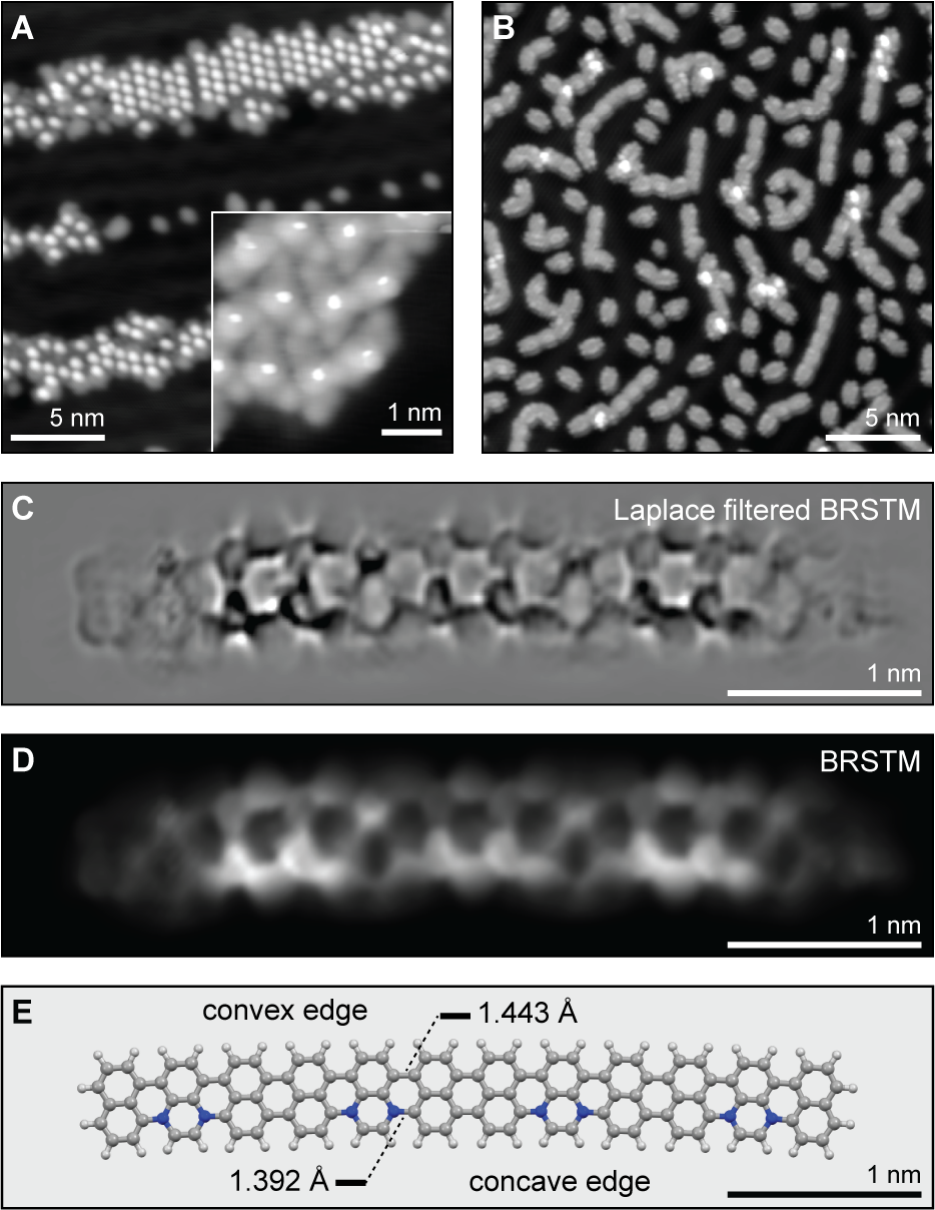
Bottom-up synthesis of N_2_-5-AGNRs. (A) STM topographic image of polymer intermediates *poly*-**1b** (*V*_s_ = −0.1 V and *I*_t_ = 80 pA). The inset shows an STM topographic image of a self-assembled island of molecular precursor **1b** on Au(111) (*V*_s_ = −0.1 V, *I*_t_ = 150 pA). (B) STM topographic images of a sample of N_2_-5-AGNRs after annealing to 310 °C (*V*_s_ = −1 V and *I*_t_ = 50 pA). (C) Laplace-filtered bond-resolved STM (BRSTM) image and (D) BRSTM image of a N_2_-5-AGNR with four repeating units (*V*_s_ = 0 V, *V*_ac_ = 20 mV, and *f* = 533 Hz). (E) DFT-LDA relaxed structure of a finite N_2_-5-AGNR tetramer.

The on-surface synthesis of N_2_-5-AGNRs from molecular precursor relies heavily on the subtle differences in the activation barrier for brominated and iodinated precursors, **1a** and **1b**, respectively.^35,36^ The lower bond dissociation energy (BDE) of C–I bonds ensures that the step growth polymerization proceeds at a low temperature and is unaffected by trace hydrogen atoms generated during the cyclodehydrogenation step that could lead to undesired chain termination. N_2_-5-AGNRs were prepared following established surface-assisted GNR growth protocols. Molecular precursor **1b** was sublimed in ultrahigh vacuum (UHV) from a Knudsen cell evaporator onto a Au(111) surface held at *T* = 25 °C. The inset in Figure 3A shows a topographic STM image of a self-assembled island of **1b**. **1b** adopts a quasi-*C*_s_ symmetric conformation on the surface reminiscent of a crescent. A bright feature associated with the central quinoxaline ring protruding from the surface is flanked on either side by iodonaphthalene units adsorbed nearly coplanar to the underlying substrate.

Annealing the molecule-decorated surface at *T*_1_ = 100 °C for 30 min induces a radical step-growth polymerization that gives rise to polymer intermediate *poly*-**1b** (Figure 3A). A second annealing step at *T*_2_ = 310 °C for 20 min induces a cyclodehydrogenation reaction that leads to the formation of two C–N bonds and one C–C bond per unit cell. Topographic STM images recorded on a low coverage sample (Figures 3B and S1A–C) are dominated by small rectangular structures consistent with the dimension of fully fused monomers (1.2 × 0.68 × 0.18 nm). These smaller molecular structures are interspersed by linear oligomeric nanoribbons featuring a characteristic pattern of periodic constrictions and ranging in length from the dimer (∼2 nm) to the hexamer (∼6 nm). Bond-resolved STM (BRSTM) with a CO-functionalized tip recorded on a tetramer reveals the characteristic internal bonding associated with 5-AGNRs (Figure 3C). An alternating pattern of dark and bright low-bias features is seen along the backbone of the ribbon (Figure 3D). Regions of brighter contrast correspond to 5-AGNR segments resulting from the fusion of two naphthalene units (the perylene core), while the darker features coincide with the position of the N atoms (the quinoxaline core). The subtle curvature of N_2_-5-AGNRs suggests a preference for the alignment of N atoms along one armchair edge (the bottom edge of the ribbon depicted in Figure 3C,D). Figure 3E shows the calculated C–N and C–C bond lengths for a N_2_-5-AGNR tetramer of 1.392 and 1.443 Å, respectively. The periodic bond distortion along the main axis of the ribbon gives rise to the distinctive structure of coexisting convex (all-carbon) and concave (N atom doped) armchair edges. Bond length alternations have previously been observed in other pyrazine-embedded nanographene structures.^31,37^

### Electronic Structure Characterization of N_2_-5-AGNRs

**Figure 4:**
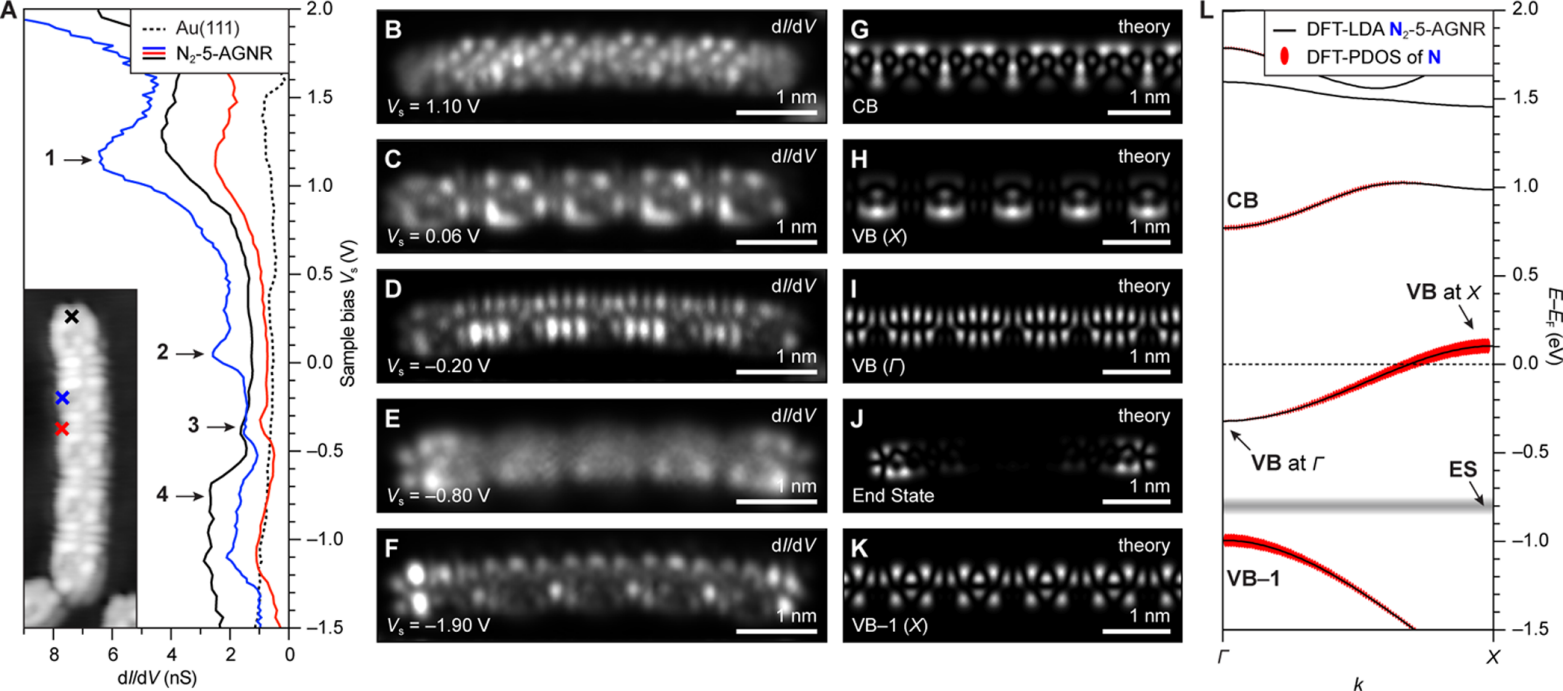
Electronic structure of N_2_-5-AGNRs. (A) STS d*I*/d*V* spectra recorded on a N_2_-5-AGNR with five repeating units at the marked positions in the inset STM topographic image. Prominent features in the d*I*/d*V* spectra are labeled as *Peak* 1–4 (spectroscopy: *V*_ac_ = 4 mV, *f* = 533 Hz; imaging: *V*_s_ = −0.1 V, *I*_t_ = 100 pA, CO-functionalized tip). (B–F) Constant height d*I*/d*V* maps recorded at the indicated biases (*V*_ac_ = 20 mV, *f* = 533 Hz, and a CO-functionalized tip). (G–I, K) DFT-simulated LDOS maps of the CB bottom, VB top, VB bottom, and VB–1 bottom of N_2_-5-AGNR. The maps are calculated at the band edge energies. (J) DFT-simulated LDOS map of the end-states of N_2_-5-AGNR with five repeating units. (L) DFT-LDA-calculated band structure of N_2_-5-AGNR. Features associated with the CB, VB, and ES are indicated by arrows. *E*_F_ location is inferred through comparison of spectroscopic GNR features and Au(111) *E*_F_ location (see text).

We characterized the electronic structure of N_2_-5-AGNRs using STM spectroscopy. Figure 4A shows typical d*I*/d*V* spectra of a N_2_-5-AGNR recorded at the positions marked in the inset. Four prominent spectral features appear in the range of −1.5 V < *V*_s_ < + 1.5 V. Two peaks centered at *V*_s_ = +1.10 V (*Peak* 1) and *V*_s_ = +0.06 V (*Peak* 2) are most prominent, along with a shoulder at *V*_s_ = −0.4 V (*Peak* 3). The intensities of *Peak* 1 and 2 are largest when the STM tip is placed above the position of a quinoxaline N atom (blue line in Figure 4A). *Peak* 3 is most prominent in spectra recorded above the position of the fused naphthalene units (red line in Figure 4A) and the positions of N atoms. The last spectral feature, a shoulder at *V*_s_ = −0.75 V (*Peak* 4), is most prominent near the zigzag end of the N_2_-5-AGNRs (black line in Figure 4A).

Differential conductance maps recorded at or near the energies of *Peaks* 1–4 (Figure 4B–E) provide further insight into the spatial distribution of the wave function associated with the corresponding electronic states. The d*I*/d*V* map at *V*_s_ = +1.10 V (*Peak* 1, Figure 4B) shows a distinctive nodal pattern of bright spots emanating from the convex armchair edge that decays across the width of the ribbon and vanishes toward the zigzag ends. A similar characteristic pattern can be observed in a d*I*/d*V* map acquired at *V*_s_ = −1.90 V (Figures 4F and S2). The latter map exhibits a pair of bright lobes at either end of the ribbon. Differential conductance maps at a bias associated with *Peak* 2 (*V*_s_ = +0.06 V; Figure 4C) reveal a nodal pattern that clearly outlines the crystallographic unit cells of a N_2_-5-AGNR pentamer. Each cell is bounded by a U-shaped bright feature along the concave armchair edge (commensurate with the position of the quinoxaline N atoms) matched on the convex side by two fainter lobes. As the mapping bias is swept across *E*_F_, the nodal pattern evolves into four prominent lobes along the concave edge at *V*_s_ = −0.20 V (*Peak* 3, Figure 4D). These are associated with the fused naphthalene units that are mirrored, albeit at a lower signal intensity, across the width of the ribbon. Finally, d*I*/d*V* maps recorded at *V*_s_ = −0.80 V (*Peak* 4, Figure 4E) show the characteristic signature of a localized N_2_-5-AGNR end-state.

### First-Principles Calculation of N_2_-5-AGNR LDOS

*Ab initio* DFT calculations enable the rationalization of the spectral features revealed in STS experiments. Figure 4G–K shows the calculated LDOS maps evaluated at the N_2_-5-AGNR CB minimum, the top and bottom VB edges, the end-states, and the VB–1 edge, respectively. The similarity between theoretically predicted nodal patterns associated with the CB minimum (Figure 4G) and the experimental d*I*/d*V* map recorded at *V*_s_ = +1.10 V is striking and suggests an assignment of *Peak* 1 to the CB edge. The evolution of the calculated LDOS sampled at the VB top at ***k*** = *X* (Figure 4H) and the VB bottom at ***k*** = *Γ* (Figure 4I) is also mirrored in the experimental d*I*/d*V* maps recorded at *V*_s_ = +0.06 V (*Peak* 2, Figure 4C) and *V*_s_ = −0.2 V (*Peak* 3, Figure 4D), respectively. The gradual increase of density from orbitals with N atom character as ***k*** is swept from *Γ* to *X* in the theoretical LDOS is reflected in the increased intensity in d*I*/d*V* maps around N atoms as the bias is shifted from *V*_s_ = +0.06 V (Figure 4C) to *V*_s_ = −0.20 V (Figure 4D). Similarly, the theoretical LDOS maps associated with the end-states of a finite N_2_-5-AGNR (Figure 4J) and the VB–1 edge at ***k*** = *X* (Figure 4K) are in good agreement with the experimental maps recorded at *V*_s_ = −0.80 V (Figure 4E) and *V*_s_ = −1.90 V (Figure 4F).

The picture that emerges from a comparison between theory and experiment for the electronic structure of N_2_-5-AGNRs on Au(111) is shown in Figure 4L. To match the energy levels of valence band onsets between experimental DOS in Figure 4A and theoretical DOS in Figure 4L, the DFT-calculated band structure of Figure 1C has been rigidly shifted up in energy so that *E*_F_ lies 0.1 eV below the VB maximum to model hole-doping from the Au(111) substrate (see Supporting Information Figure S3 for calculated DOS). While DFT-LDA calculations are known to underestimate the size of the band gap, the gold substrate itself introduces a screening of the GNR that partially compensates for the underestimation of the band gap by DFT-LDA. The characteristic topological end-state of a finite, all-carbon 5-AGNR that would normally lie at *E*_F_ is now found 0.8 eV below *E*_F_ (i.e., *Peak* 4) due to the additional electron density arising from the N-dopants. This is consistent with the close match between the experimental d*I*/d*V* maps of Figure 4B–F and the theoretical LDOS distributions shown in Figure 4G–K. The lack of any observable end-states in the energy range between the VB maximum and CB minimum is also consistent with the change in topological character in N_2_-5-AGNRs from nontrivial (in all-carbon 5-AGNRs) to trivial.

### Lift-Off and Charge Transport Experiments

**Figure 5:**
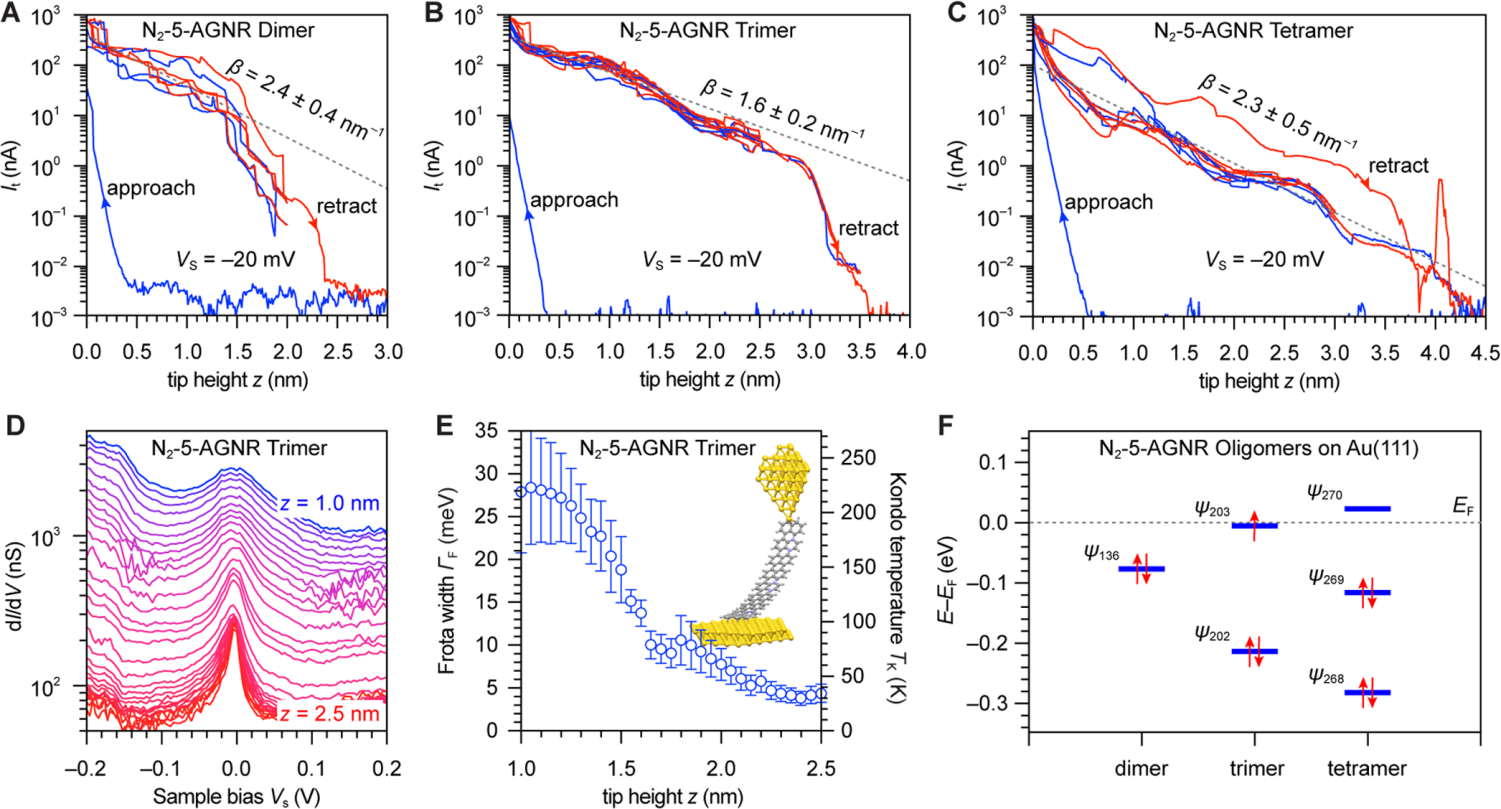
N_2_-5-AGNR lift-off experiments. Plot of the tunneling current (*I*_t_) vs. relative tip height (*z*) for the lifting of a N_2_-5-AGNR (A) dimer, (B) trimer, and (C) tetramer at *V*_s_ = −20 mV. Color coding: retraction of the STM tip (red), approach of the STM tip (blue). The dashed gray line represents a least-squares fit of the data to exponential decay function *I*(*z*) = *I*_0_e^–β*z*^. (D) STS lift-off experiments performed on a N_2_-5-AGNR trimer. Color gradient indicates the apparent *z*-height of the STM tip above the surface. (E) Frota fit width and Kondo temperature vs. tip height. (F) DFT-calculated molecular orbital energy-level diagram of N_2_-5-AGNR dimer, trimer, and tetramer. The dashed lines indicate the position of the *E*_F_ of a N_2_-5-AGNR on Au(111).

To gain insight into how the electronic structure of a N_2_-5-AGNR affects its electronic transport behavior, we performed lift-off through-conductance experiments. Here, one end of a N_2_-5-AGNR is attached to the STM tip and gradually lifted from the surface while recording the change in tunneling current (*I*_t_) at a constant sample bias (*V*_s_ = −20 mV).^38−40^Figure 5A–C shows the evolution of *I*_t_ as a function of tip height (*z*) for a N_2_-5-AGNR dimer, trimer, and tetramer, respectively. Each trace shows that within a narrow window defined by the length of the ribbon, *I*_t_ decays exponentially with lifting height. The large hysteresis seen for all three lengths indicates that the end of the tip is no longer in contact with the GNR. Since the energy levels of oligomeric N_2_-5-AGNRs are discrete, a resonant conduction path at this small bias voltage is unlikely.^38,41^ Transport through these short ribbons is therefore described as off-resonant tunneling with a characteristic current decay described by *I*(*z*) = *I*_0_e^–*βz*^, where *z* = 0 nm is the reference height, where *I*_0_ = 700 nA. A fit of the exponential decay function *I*(*z*) to the experimental data in Figure 5A–C (dashed gray lines) reveals characteristic tunneling decay constants (β) for the dimer (2.4 ± 0.4 nm^–1^), trimer (1.6 ± 0.2 nm^–1^), and tetramer (2.3 ± 0.5 nm^–1^) (see Supporting Information, Figure S4, for monomer data). The β-values for the dimer and tetramer are similar in magnitude, but the decay constant for trimers was consistently smaller and warranted further investigation.

Figure 5D shows a series of d*I*/d*V*(*V*) spectra recorded on a N_2_-5-AGNR trimer. A broad spectral feature centered at *V*_s_ = 0.0 V is seen to evolve into a single narrow peak as the tip is retracted from *z* = 1.0 to 2.5 nm. The *z*-independent position of the peak at zero bias and the narrowing of the peak width with lifting height provide evidence that this feature is a Kondo resonance.^32,42−44^ Here, a magnetic moment (e.g., an unpaired electron) hosted by the GNR is screened by itinerant electrons in the Au(111) surface, giving rise to zero-energy spin-flip excitations reflected in the increased d*I*/d*V* signal at *V*_s_ = 0.0 V.^45,46^ A conventional resonance peak would likely shift away from zero during the highly perturbative lift-off procedure. No analogous features were observed in d*I*/d*V* spectra recorded on N_2_-5-AGNR dimers and tetramers (Supporting Information, Figure S5).^32,39^ The curves in Figure 5D were fitted by the Frota function:^47,48^
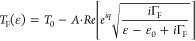
1where *T*_0_ is the background tunneling signal, *A* is the amplitude, *q* is the symmetry factor of the Frota function, ε_0_ is the center of the resonance, and Γ_F_ is the Frota parameter (0.686 Γ_F_ ∼ *k*_B_*T*_K_). These fits (shown in Figure 5E) reveal a steady decrease of *T*_K_ from 220 to 30 K as the tip is retracted from *z* = 1.0 to 2.5 nm.^47,48^ This decrease can be explained by reduced screening of the unpaired electron spin by the underlying Au substrate as the ribbon is lifted off the surface.^31^

The experimental observation of an unpaired electron in N_2_-5-AGNR trimers explains the smaller tunneling decay constant β since a secondary tunneling path through the Kondo resonance contributes to the overall through-conductance in this case.^32^ The presence of a partially filled state on the N_2_-5-AGNR trimer likely arises from a charging effect of the underlying Au substrate. Figure 5F shows the DFT-calculated molecular frontier orbital energies of a N_2_-5-AGNR dimer, trimer, and tetramer (here, *E*_F_ is derived from Figure 4L). Coincidentally, *E*_F_ is seen to align with the highest occupied molecular orbital (HOMO) energy level of the N_2_-5-AGNR trimer (Supporting Information Figure S6). This energy correlation facilitates partial charge transfer from the N_2_-5-AGNR trimer to the underlying Au substrate. Although there is some uncertainty in the precise location of *E*_F_, the observed Kondo resonance is consistent with the orbital picture presented in Figure 5F. Here, the HOMO of the dimer is low enough in energy that it is doubly occupied and thereby nonmagnetic. The tetramer is a similarly closed shell. The alignment of a trimer molecular orbital with *E*_F_, on the other hand, gives rise to a singly occupied molecular orbital (SOMO) that hosts an unpaired electron spin and is able to form a Kondo singlet due to exchange coupling with itinerant electrons in the Au(111).

## Conclusions

Fermi-level engineering has been shown to be a versatile tool for inducing pronounced changes in the band structure and topology of GNRs. Molecular building blocks can be induced to undergo regiocontrolled self-assembly to yield N_2_-5-AGNRs featuring a superlattice of N atom dopants along only one of the extended armchair edges. First-principles DFT-LDA calculations suggest that N-doping induces a sizable shift in the Fermi energy (∼1.0 eV) and is accompanied by a change of the band structure from a direct to an indirect gap semiconductor. Hole-doping associated with the interaction of N_2_-5-AGNRs and the underlying Au(111) substrate only partially counteracts the effective shift of *E*_F_ seen in experiments. Scanning tunneling spectroscopy and lift-off charge transport independently corroborate the overall theoretical picture. This work paves the way toward the realization of atomically defined band alignments at molecular-scale heterojunction and metal–semiconductor interfaces, critical components for any future molecular-scale technology.

## Experimental Section

### Materials and Instrumentation

Unless otherwise stated, all manipulations of air- and/or moisture-sensitive compounds were carried out in oven-dried glassware under an atmosphere of Ar. All solvents and reagents were purchased from Alfa Aesar, Spectrum Chemicals, Acros Organics, TCI America, and Sigma-Aldrich and used as received unless otherwise noted. Organic solvents were dried by passing through a column of alumina and were degassed by vigorous bubbling of N_2_ through the solvent for 20 min. Flash column chromatography was performed on SiliCycle silica gel (particle size 40–63 μm). Thin-layer chromatography was carried out using SiliCycle silica gel 60 Å F-254 precoated plates (0.25 mm thick) and visualized by UV absorption. All ^1^H and ^13^C NMR spectra were recorded on Bruker AV-600, MHz spectrometers, and are referenced to residual solvent peaks (CDCl_3_^1^H NMR = 7.26 ppm, ^13^C NMR = 77.16 ppm). High-resolution ESI mass spectrometry was performed on a Finnigan LTQFT (Thermo) mass spectrometer in positive ionization mode. High-resolution EI mass spectrometry was performed on an AutoSpec Premier (Waters) mass spectrometer in positive ionization mode. X-ray crystallography was performed on a Rigaku XtaLAB P200 equipped with a MicroMax 007HF dual-source rotating anode and a Pilatus 200 K hybrid pixel array detector. Data were collected using Mo Kα (λ = 0.71073 Å) radiation. Crystals were held at 100 K throughout the collection using an Oxford Cryostream 700. Data collection was performed with CrysAlisPro.^49^ Data were processed with CrysAlisPro and includes a multiscan absorption correction applied using the SCALE3 ABSPACK scaling algorithm within CrysAlisPro. Crystallographic data was solved with ShelXT, refined with ShelXL, and finalized in Olex1.5. 4,7-Diiodobenzo[*c*][1,2,5]thiadiazole (**2**) was prepared following literature procedures.^34^

#### 5,8-Diiodoquinoxaline (**3**)

A 250 mL two-neck round-bottom flask was charged with 4,7-diiodobenzo[*c*][1,2,5]thiadiazole **2** (1.30 g, 3.35 mmol) in EtOH (60 mL) and THF (20 mL). A reflux condenser was attached, and the reaction mixture was degassed by sparging with N_2_ for 20 min. NaBH_4_ (0.380 g, 10.1 mmol) was slowly added to the reaction mixture in small portions. CoCl_2_·6H_2_O was added (0.008 g, 0.034 mmol), and the reaction mixture was stirred for 8 h at 78 °C. The reaction mixture was concentrated, diluted with H_2_O (50 mL), and extracted with Et_2_O (100 mL). The combined organic phases were washed with H_2_O (50 mL) and saturated aqueous NaCl (100 mL), dried over MgSO_4_, and concentrated on a rotary evaporator. Crude 3,6-diiodobenzene-1,2-diamine (1.21 g) was used without further purification. A 100 mL two-neck round-bottom flask was charged with 3,6-diiodobenzene-1,2-diamine (1.21 g, 3.36 mmol) in EtOH (50 mL). A reflux condenser was attached, and the reaction mixture was degassed by sparging with N_2_ for 20 min. Glyoxal (610 μL, 4.20 mmol, 40 wt % solution in H_2_O) was added dropwise, and the reaction mixture was stirred under a N_2_ atmosphere for 24 h at 78 °C. The reaction mixture was concentrated, diluted with H_2_O (100 mL), and extracted with CHCl_3_ (200 mL). The combined organic phases were washed with H_2_O (50 mL), saturated aqueous NaCl (100 mL), dried over MgSO_4_, and concentrated on a rotary evaporator. Column chromatography (SiO_2_; 8:1:1 hexane/CH_2_Cl_2_/EtOAc) yielded **3** (0.077 g, 0.202 mmol, 6%) as a yellow solid. ^1^H NMR (600 MHz, CDCl_3_) δ = 8.91 (s, 2H), 8.13 (s, 2H) ppm; ^13^C {^1^H} NMR (151 MHz, CDCl_3_) δ = 146.7, 143.3, 141.4 ppm; HRMS (EI-TOF) *m*/*z*: [C_8_H_4_I_2_N_2_]^+^ calcd [C_8_H_4_I_2_N_2_]^+^ 381.8464; found, 381.8468.

#### 2-(4-Bromonaphthalen-1-yl)-4,4,5,5-tetramethyl-1,3,2-dioxaborolane (**4**)

A 250 mL two-neck round-bottom flask was charged under N_2_ with 1,4-dibromonaphthalene (3.00 g, 10.5 mmol) in dry THF (80 mL). The reaction mixture was cooled to −78 °C and stirred for 20 min. *n*-BuLi (5.04 mL, 12.6 mmol, 2.5 M in hexanes) was added dropwise, and the reaction mixture was stirred for 1 h at −78 °C. 2-Isopropoxy-4,4,5,5-tetramethyl-1,3,2-dioxaborolane (3.00 mL, 14.7 mmol) was added dropwise, and the reaction mixture was stirred for 8 h at −78 °C. The reaction mixture was quenched by the addition of 2 M HCl (10 mL), diluted with H_2_O (100 mL), and extracted with EtOAc (200 mL). The combined organic phases were washed with H_2_O (100 mL) and saturated aqueous NaCl (100 mL), dried over Na_2_SO_4_, and concentrated on a rotary evaporator. Column chromatography (SiO_2_; 9:1:0.01 hexane/CH_2_Cl_2_/EtOAc) yielded **4** (3.11 g, 9.34 mmol, 89%) as a colorless crystalline solid. ^1^H NMR (600 MHz, CDCl_3_) δ = 8.80–8.75 (m, 1H), 8.31–8.25 (m, 1H), 7.89 (d, *J* = 7.4 Hz, 1H), 7.79 (s, 1H), 7.61–7.55 (m, 2H), 1.42 (s, 13H) ppm; ^13^C {^1^H} NMR (151 MHz, CDCl_3_) δ = 138.3, 135.8, 131.9, 129.5, 129.0, 127.5, 127.4, 127.3, 127.1, 84.1, 25.1 ppm; HRMS (EI-TOF) *m*/*z*: [C_16_H_18_BBrO_2_]^+^ calcd [C_16_H_18_BBrO_2_] 334.0563; found, 334.0563.

#### 5,8-Bis(4-bromonaphthalen-1-yl)quinoxaline (**1a**)

A 25 mL Schlenk tube was charged with **3** (0.050 g, 0.131 mmol), **4** (0.089 g, 0.269 mmol), bis(triphenylphosphine)palladium(II) dichloride (0.009 g, 0.013 mmol), and Na_2_CO_3_ (0.056 g, 0.524 mmol) in dioxane (5 mL) and H_2_O (2 mL). The reaction mixture was degassed by sparging with N_2_ for 20 min and stirred under N_2_ for 18 h at 80 °C. The reaction mixture was diluted with H_2_O (20 mL) and extracted with CH_2_Cl_2_ (70 mL). The combined organic phases were washed with H_2_O (50 mL) and saturated aqueous NaCl (50 mL), dried over Na_2_SO_4_, and concentrated on a rotary evaporator. Column chromatography (SiO_2_; 1:1:0.1 hexane/CH_2_Cl_2_/EtOAc) yielded **1a** (0.056 g, 0.103 mmol, 79%) as a yellow solid. ^1^H NMR (600 MHz, CDCl_3_) δ = 8.73 (s, 2H), 8.45–8.32 (m, 2H), 7.97 (t, *J* = 7.7 Hz, 2H), 7.93 (s, 2H), 7.63 (dddd, *J* = 11.3, 8.3, 6.6, 1.3 Hz, 2H), 7.55–7.52 (m, 2H), 7.49 (d, *J* = 7.5 Hz, 2H), 7.47–7.39 (m, 4H) ppm; ^13^C {^1^H} NMR (151 MHz, CDCl_3_) δ = 145.1, 145.1, 142.1, 142.1, 139.9, 139.9, 136.8, 136.8, 134.1, 134.1, 132.2, 132.2, 131.6, 131.5, 129.6, 129.6, 128.7, 128.6, 127.8, 127.8, 127.4, 127.4, 127.1, 127.1, 127.0, 127.0, 123.7, 123.6 ppm; HRMS (EI-TOF) *m*/*z*: [C_28_H_16_Br_2_N_2_]^+^ calcd [C_28_H_16_Br_2_N_2_] 539.9660; found 539.9656.

#### 5,8-Bis(4-iodonaphthalen-1-yl)quinoxaline (**1b**)

A 10 mL Schlenk tube was charged under N_2_ with **1a** (0.042 g, 0.078 mmol), NaI (0.089 g, 0.777 mmol), CuI (0.009 g, 0.016 mmol), and racemic *trans*-*N*,*N*′-dimethyl-1,2-cyclohexanediamine (2.5 μL, 0.02 mmol) in dry, degassed dioxane (2 mL). The Schlenk tube was sealed with a Teflon valve, and the reaction mixture was stirred under N_2_ for 24 h at 110 °C. The reaction mixture was diluted with H_2_O (10 mL) and extracted with EtOAc (30 mL). The combined organic phases were washed with H_2_O (10 mL) and saturated aqueous NaCl (10 mL), dried over Na_2_SO_4_, and concentrated on a rotary evaporator. Column chromatography (SiO_2_; 9:1:0.2 hexane/CH_2_Cl_2_/EtOAc) yielded **1b** (0.018 g, 0.029 mmol, 37%) as a colorless crystalline solid. ^1^H NMR (600 MHz, CDCl_3_) δ = 8.73 (s, 2H), 8.26 (ddd, *J* = 12.2, 8.0, 4.9 Hz, 4H), 7.92 (s, 2H), 7.65–7.54 (m, 2H), 7.54–7.28 (m, 6H) ppm; ^13^C {^1^H} NMR (151 MHz, CDCl_3_) δ = 145.1, 145.1, 142.1, 139.9, 139.9, 137.1, 137.1, 134.4, 134.4, 133.6, 132.9, 131.5, 129.3, 129.2, 127.9, 127.8, 127.2, 127.2, 127.1, 127.0, 100.6 ppm; HRMS (ESI-TOF) *m*/*z*: [C_28_H_16_I_2_N_2_]^+^ calcd [C_28_H_16_I_2_N_2_] 634.9476; found, 634.9470.

### N_2_-5-AGNR Growth on Au(111) Surfaces

N_2_-5-AGNRs were grown on Au(111)/mica films under UHV conditions. Atomically clean Au(111) surfaces were prepared through iterative Ar^+^ sputter/anneal cycles. Submonolayer coverage of **1b** on atomically clean Au(111) was obtained by sublimation at crucible temperatures of 453–473 K using a Knudsen cell evaporator. After deposition, the surface temperature was slowly ramped (≤2 K min^–1^) to 453 K and held at this temperature for 15 min to induce the radical step growth polymerization and then slowly ramped (≤2 K min^–1^) to 623 K and held there for 15 min to induce cyclodehydrogenation.

### Scanning Tunneling Microscopy and Spectroscopy

All STM experiments were performed using a commercial Createc LT-STM operating at *T* = 4 K and using tungsten STM tips. d*I*/d*V* measurements were recorded with CO-functionalized STM tips using a lock-in amplifier with a modulation frequency of *f* = 533 Hz and a modulation amplitude of *V*_ac_ = 1.0–4.0 mV. d*I*/d*V* point spectra and d*I*/d*V* maps were recorded in the constant height mode.

### Calculations

First-principles DFT calculations in the LDA and LSDA approximations were implemented using the Quantum Espresso package.^50^ We used norm-conserving (NC) pseudopotentials with a 60 Ry energy cutoff and 0.005 Ry Gaussian broadening. To ensure the accuracy of our results, a sufficiently large vacuum region was included in the supercell calculation. All of the dangling bonds at the edges of the carbon skeleton were hydrogenated. The structures were fully relaxed until all components of the force were smaller than 0.01 eV/ Å. A periodic infinite model of N_2_-5-AGNR was used to calculate band structures in Figures 1B–C and 4L, and LDOS maps in Figure 4G–I, K. A finite-length model of a N_2_-5-AGNR was used to calculate the LDOS maps in Figure 4J and energy levels in Figure 5F.

## Supporting Information Available

Additional experimental details, STM topographic images, STS lift-off experiments, computational data, materials and methods, all NMR spectra, and X-ray crystal structure data for **1b** (PDF)
