# Supplementary material for: Fermi-Level Engineering of Nitrogen Core-Doped Armchair Graphene Nanoribbons
Source: J Am Chem Soc. 2023 Aug 23;145(35):19338–46. doi: 10.1021/jacs.3c05755 (PMC10485924; doi:10.1021/jacs.3c05755)
Supplement: Supplementary file 1 — ja3c05755_si_001.pdf [file ja3c05755_si_001.pdf]

Fermi Level Engineering of Nitrogen Core-Doped Armchair Graphene Nanoribbons

Ethan Chi Ho Wen,[†,‡] Peter H. Jacobse,[‡,‡] Jingwei Jiang,[‡,§,‡] Ziyi Wang,[‡,§] Steven G. Louie,[‡,§,*] Michael F. Crommie,[‡,§,¶,*] and Felix R. Fischer[†,§,¶,◊,*]

[†]Department of Chemistry, University of California, Berkeley, CA 94720, U.S.A.

[‡]Department of Physics, University of California, Berkeley, CA 94720, U.S.A.

[§]Materials Sciences Division, Lawrence Berkeley National Laboratory, Berkeley, CA 94720, U.S.A.

[¶]Kavli Energy NanoSciences Institute at the University of California Berkeley and the Lawrence Berkeley National Laboratory, Berkeley, California 94720, U.S.A.

[◊]Bakar Institute of Digital Materials for the Planet, Division of Computing, Data Science, and Society, University of California, Berkeley, CA 94720, USA.

**1.**	**Figure S1**. Bottom-up synthesis of N_2-5-AGNRs.	**S2**
**2.**	**Figure S2.** Electronic structure of N_2-5-AGNR.	**S3**
**3.**	**Figure S3.** DFT-LDA-calculated DOS of N_2-5-AGNR.	**S4**
**4.**	**Figure S4.** Logarithmic plot of the tunneling current (I_t) *vs.* relative tip height (z) for the lifting of a N_2-5-AGNR monomer.	**S5**
**5.**	**Figure S5.** STS lift-off experiments performed on dimer and tetramer N_2-5-AGNR.	**S6**
**6.**	**Figure S6.** DFT calculated molecular orbital energy level diagram of N_2-5-AGNR dimer, trimer, tetramer calibrated to the vacuum level.	**S7**
**7.**	**Figure S7.** ^{1}H NMR (600 MHz, $CDCl_3$) of 5,8-diiodoquinoxaline (**3**) at 24 °C.	**S8**
**8.**	**Figure S8.** ^{13}C {^{1}H} NMR (151 MHz, $CDCl_3$) of 5,8-diiodoquinoxaline (**3**) at 24 °C.	**S9**
**9.**	**Figure S9.** ^{1}H NMR (600 MHz, $CDCl_3$) of 2-(4-bromonaphthalen-1-yl)-4,4,5,5-tetramethyl-1,3,2-dioxaborolane (**4**) at 24 °C.	**S10**
**10.**	**Figure S10.** ^{13}C {^{1}H} NMR (151 MHz, $CDCl_3$) of 2-(4-bromonaphthalen-1-yl)-4,4,5,5-tetramethyl-1,3,2-dioxaborolane (**4**) at 24 °C.	**S11**
**11.**	**Figure S11.** ^{1}H NMR (600 MHz, $CDCl_3$) of 5,8-bis(4-bromonaphthalen-1-yl)quinoxaline (**1a**) at 24 °C.	**S12**
**12.**	**Figure S12.** ^{13}C {^{1}H} NMR (151 MHz, $CDCl_3$) of 5,8-bis(4-bromonaphthalen-1-yl)quinoxaline (**1a**) at 24 °C.	**S13**
**13.**	**Figure S13.** ^{1}H NMR (600 MHz, $CDCl_3$) of 5,8-bis(4-iodonaphthalen-1-yl)quinoxaline (**1b**) at 24 °C.	**S14**
**14.**	**Figure S14.** ^{13}C {^{1}H} NMR (151 MHz, $CDCl_3$) of 5,8-bis(4-iodonaphthalen-1-yl)quinoxaline (**1b**) at 24 °C.	**S15**
**15.**	**Figure S15.** Single crystal X-ray structure diagram of 5,8-bis(4-iodonaphthalen-1-yl)quinoxaline (**1b**).	**S16**
**16.**	**Table S1.** Crystal data and structure refinement for 5,8-bis(4-iodonaphthalen-1-yl)quinoxaline (**1b**).	**S16**
**17.**	**Table S2.** Atomic coordinates (× 10^4) and equivalent isotropic displacement parameters (Å^2 × 10^3) for 5,8-bis(4-iodonaphthalen-1-yl)quinoxaline (**1b**).	**S17**
**18.**	**Table S3.** Bond lengths [Å] and angles [°] for 5,8-bis(4-iodonaphthalen-1-yl)quinoxaline (**1b**).	**S19**
**19.**	**Table S4.** Anisotropic displacement parameters (Å^2 × 10^3) for 5,8-bis(4-iodonaphthalen-1-yl)quinoxaline (**1b**).	**S24**
**20.**	**Table S5.** Hydrogen coordinates (× 10^4) and isotropic displacement parameters (Å^2 × 10^3) for 5,8-bis(4-iodonaphthalen-1-yl)quinoxaline (**1b**).	**S26**
**21.**	**Table S6.** Torsion angles [°] for 5,8-bis(4-iodonaphthalen-1-yl)quinoxaline (**1b**).	**S27**

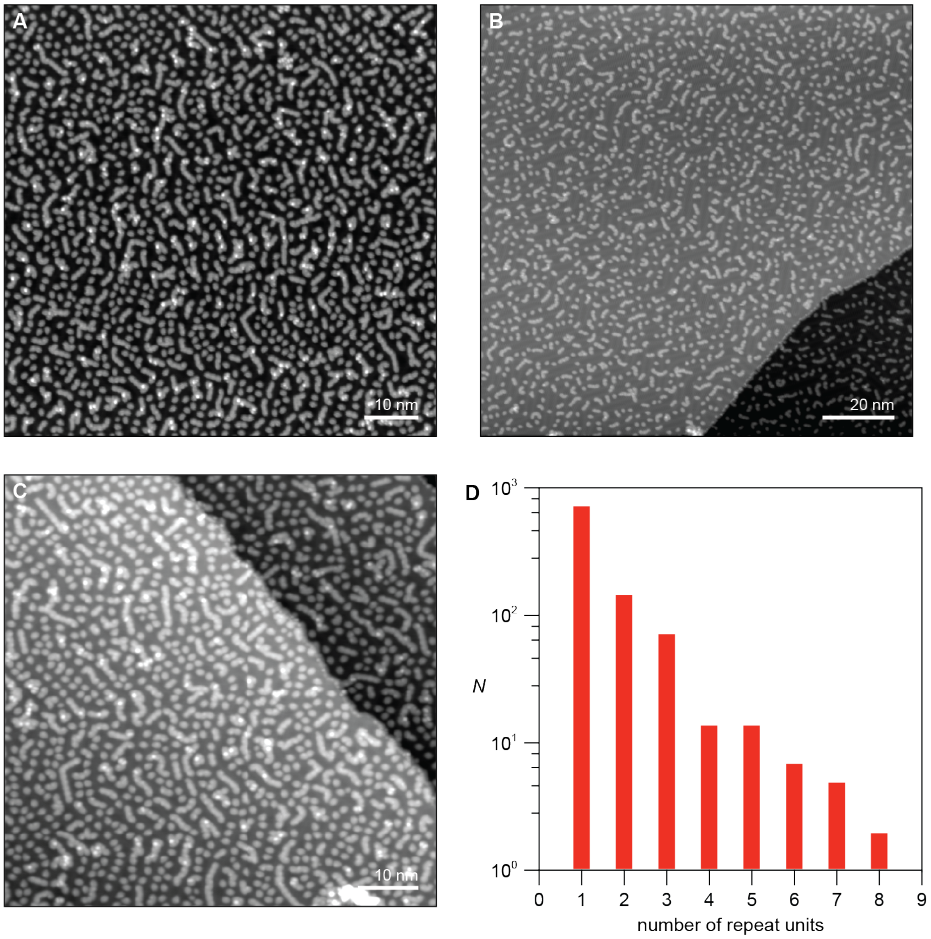

Figure S1. Bottom-up synthesis of N_2-5-AGNRs. (A-C) STM topographic images of N_2-5-AGNRs following annealing to 310 °C (V_s = –800 mV, I_t = 50 pA). (D) Statistical distribution of N_2-5-AGNR lengths grown from a sub-monolayer (ML) sample of precursor **1b**. Data is sampled from Figure 3B and Figure S1C.

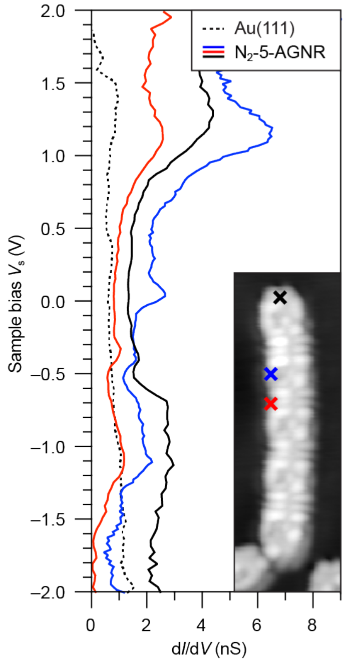

Figure S2. Electronic structure of N_2-5-AGNR. Extended STS dI/dV spectra recorded on a N_2-5-AGNR with five repeating units at the marked positions in the inset STM topographic image (spectroscopy: V_{ac} = 4 mV, f = 533 Hz; imaging: V_s = –0.1 V, I_t = 100 pA, CO-functionalized tip).

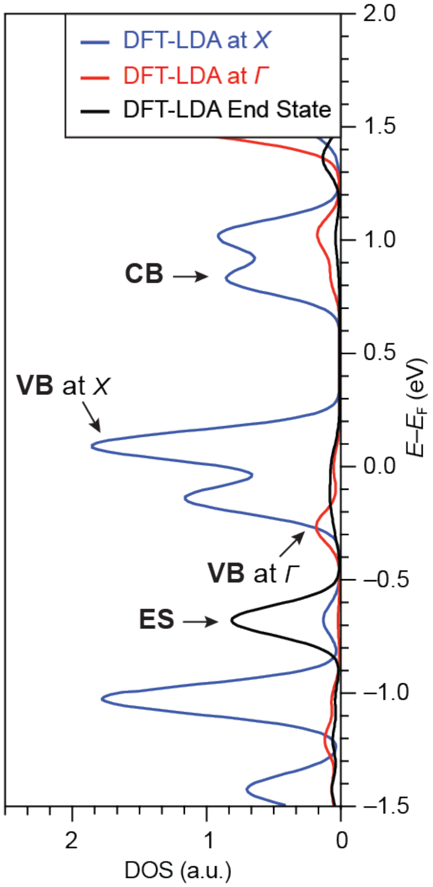

Figure S3. DFT-LDA-calculated DOS of N_2-5-AGNR with five repeating units. The scale has been rigidly shifted up in energy so that E_F lies 0.1 eV below the VB maximum to model hole-doping from the Au(111) substrate (spectrum broadened by 10 meV Gaussian).

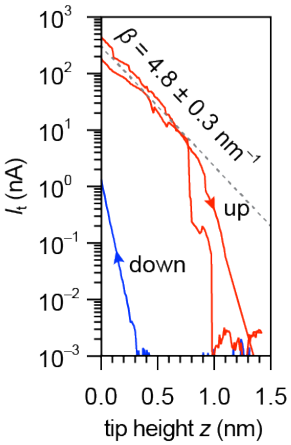

Figure S4. Plot of the tunneling current (I_t) *vs*. relative tip height (z) for the lifting of a N_2-5-AGNR monomer at –20 mV.

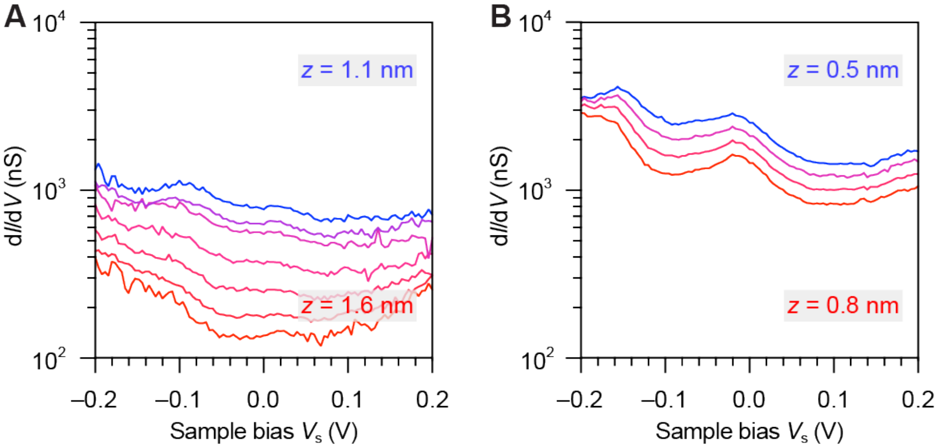

Figure S5. STS lift-off experiments performed on (A) dimer and (B) tetramer N_2-5-AGNR. Color gradient indicates the apparent z-height of the STM tip above the surface.

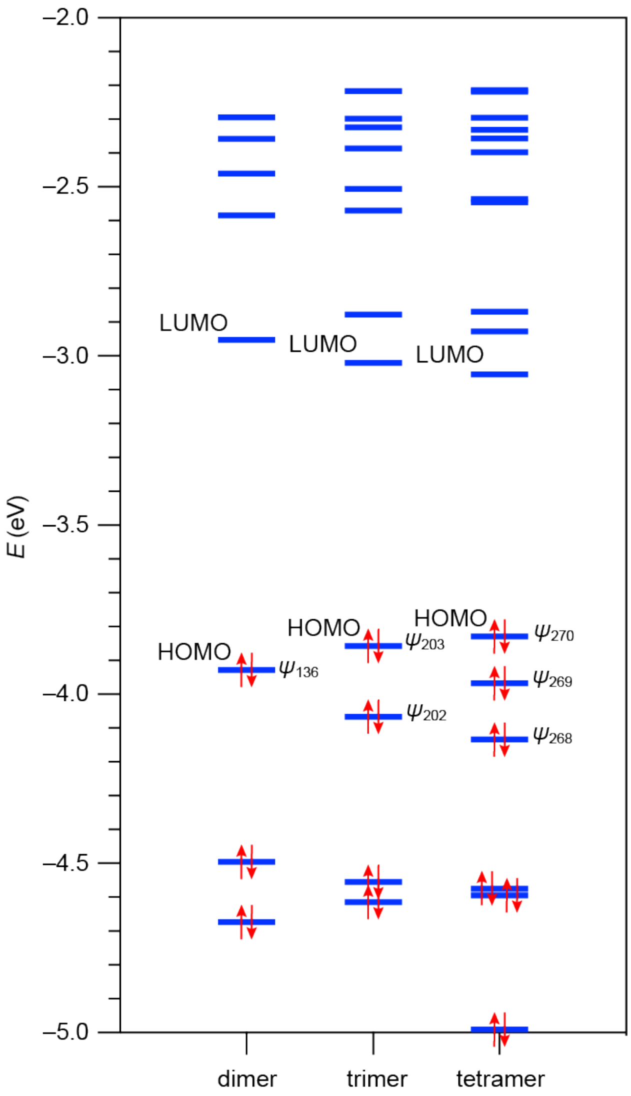

Figure S6. DFT-LDA calculated molecular orbital energy level diagram of N_2-5-AGNR dimer, trimer, tetramer calibrated to the vacuum level energy.

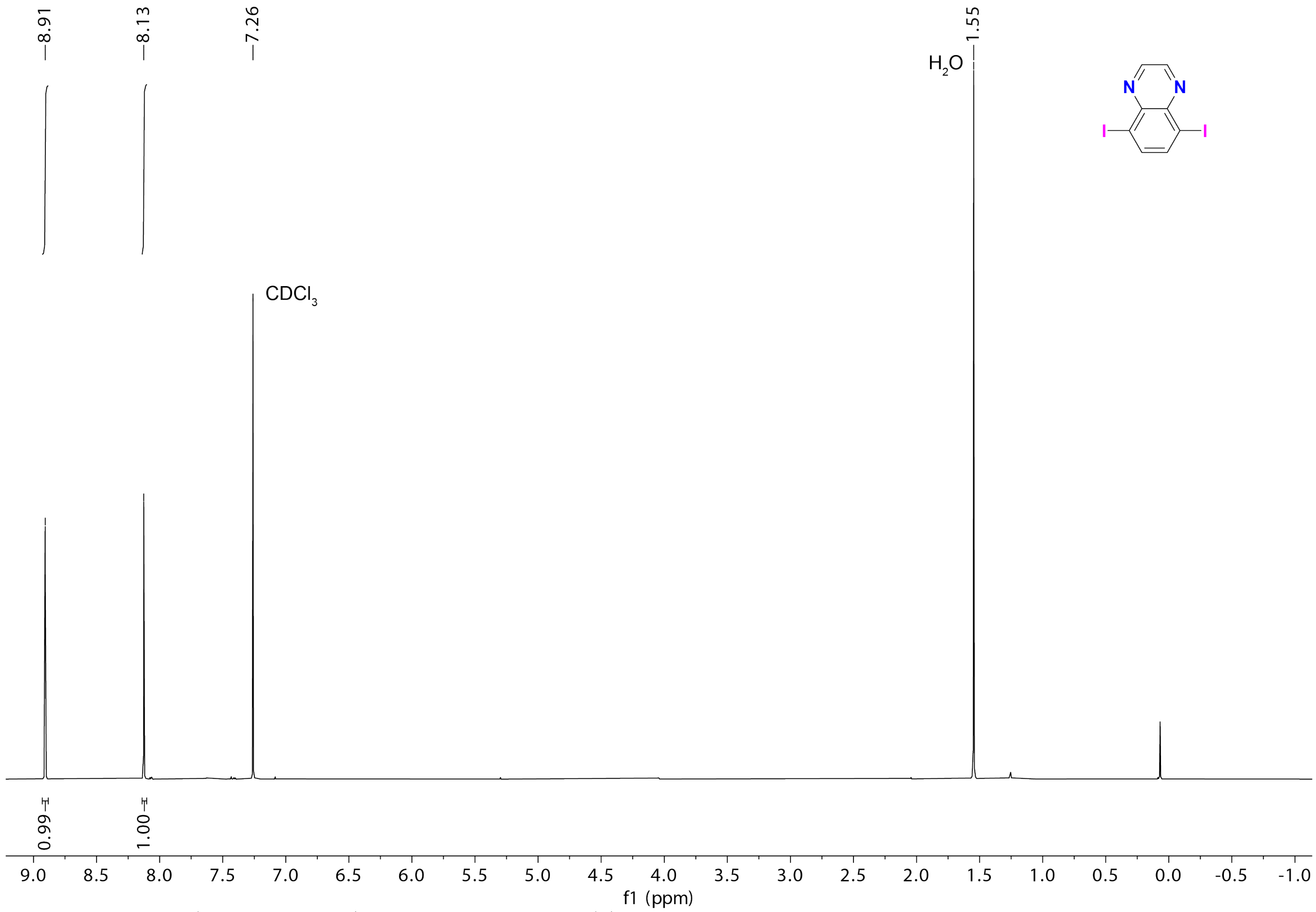

Figure S7. ^{1}H NMR (600 MHz, $CDCl_3$) of 5,8-diiodoquinoxaline (**3**) at 24 °C.

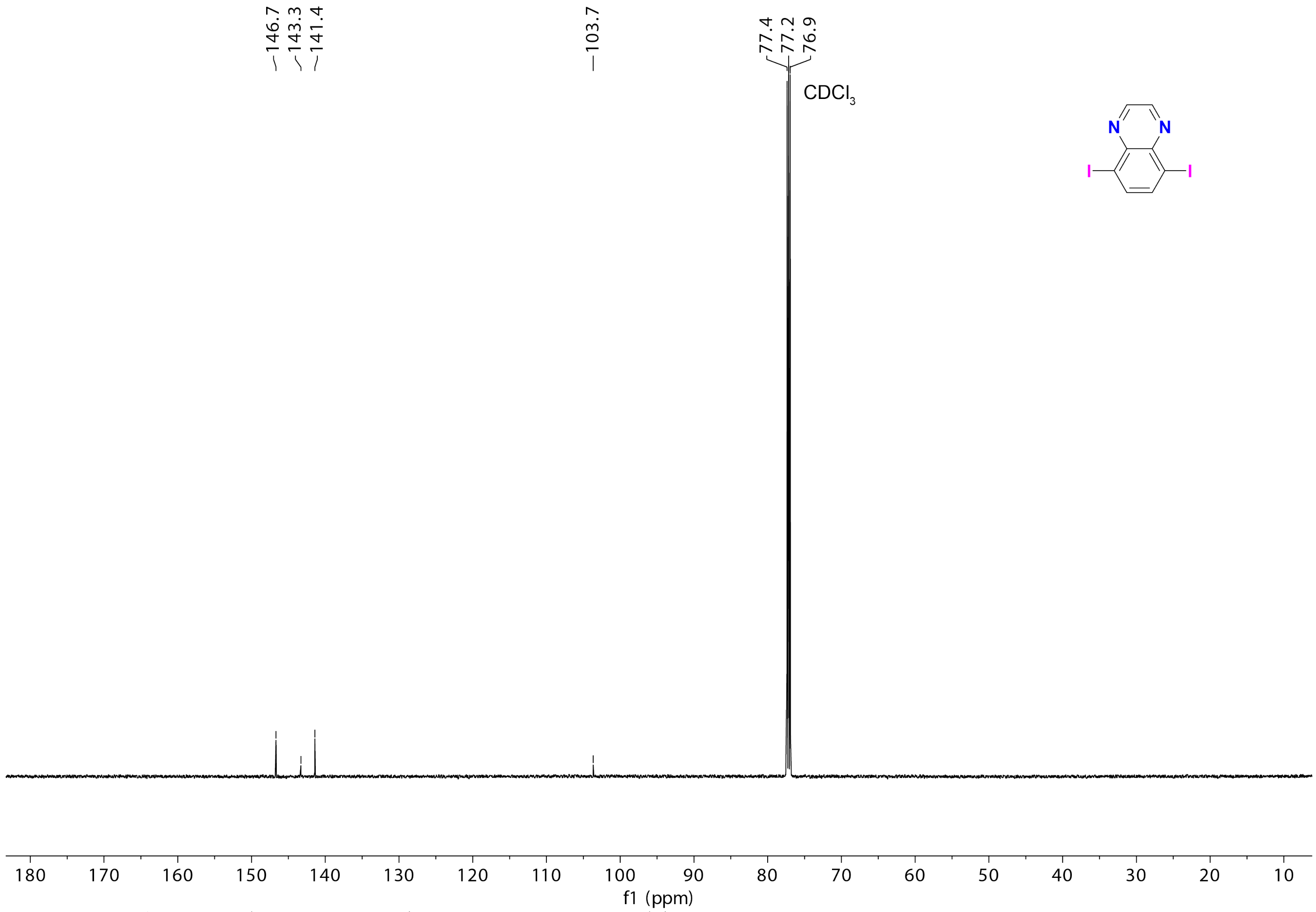

Figure S8. $^{13}C\{^1H\}$ NMR (151 MHz, $CDCl_3$) of 5,8-diiodoquinoxaline (**3**) at 24 °C.

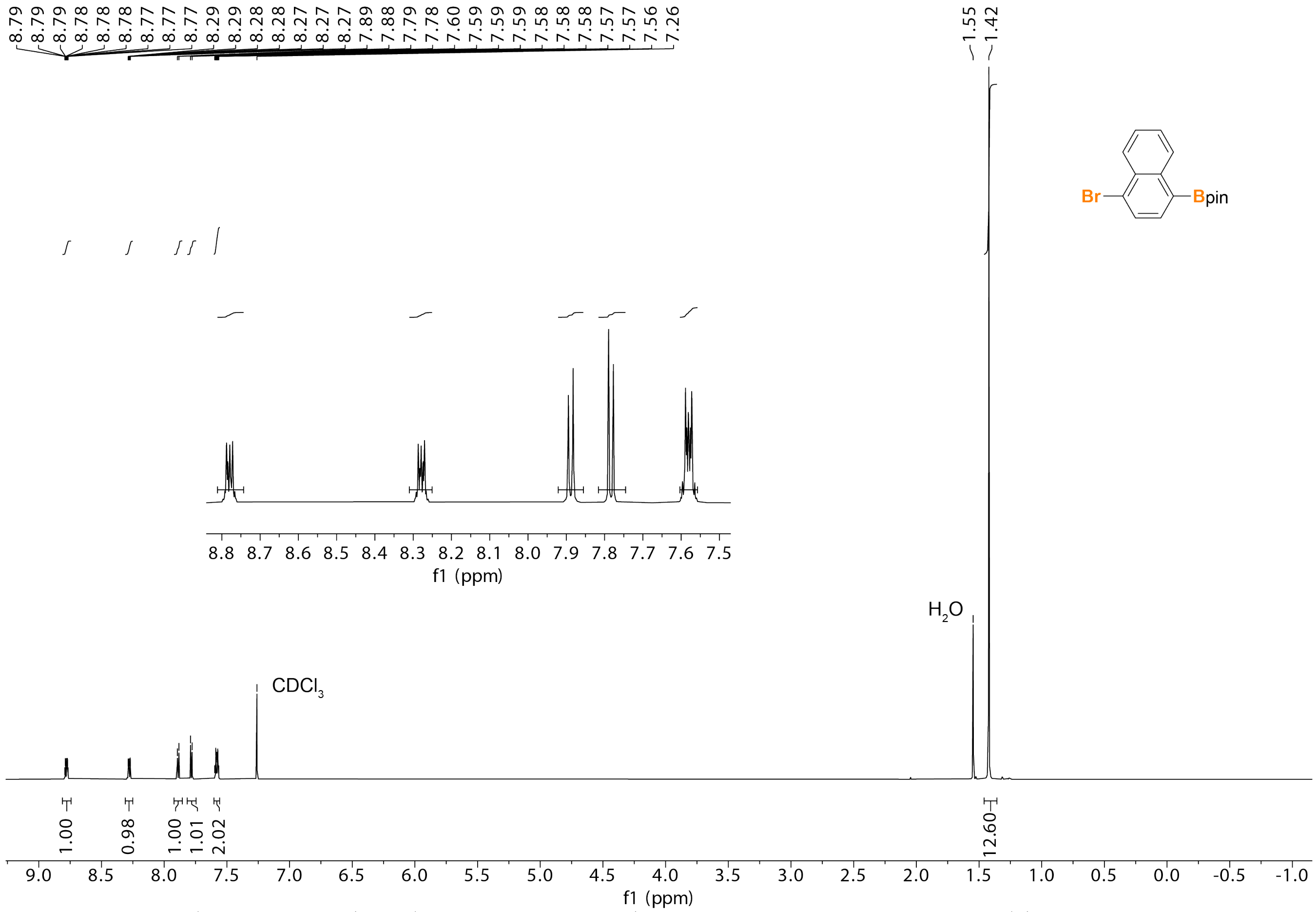

Figure S9. ^1H NMR (600 MHz, $CDCl_3$) of 2-(4-bromonaphthalen-1-yl)-4,4,5,5-tetramethyl-1,3,2-dioxaborolane (**4**) at 24 °C.

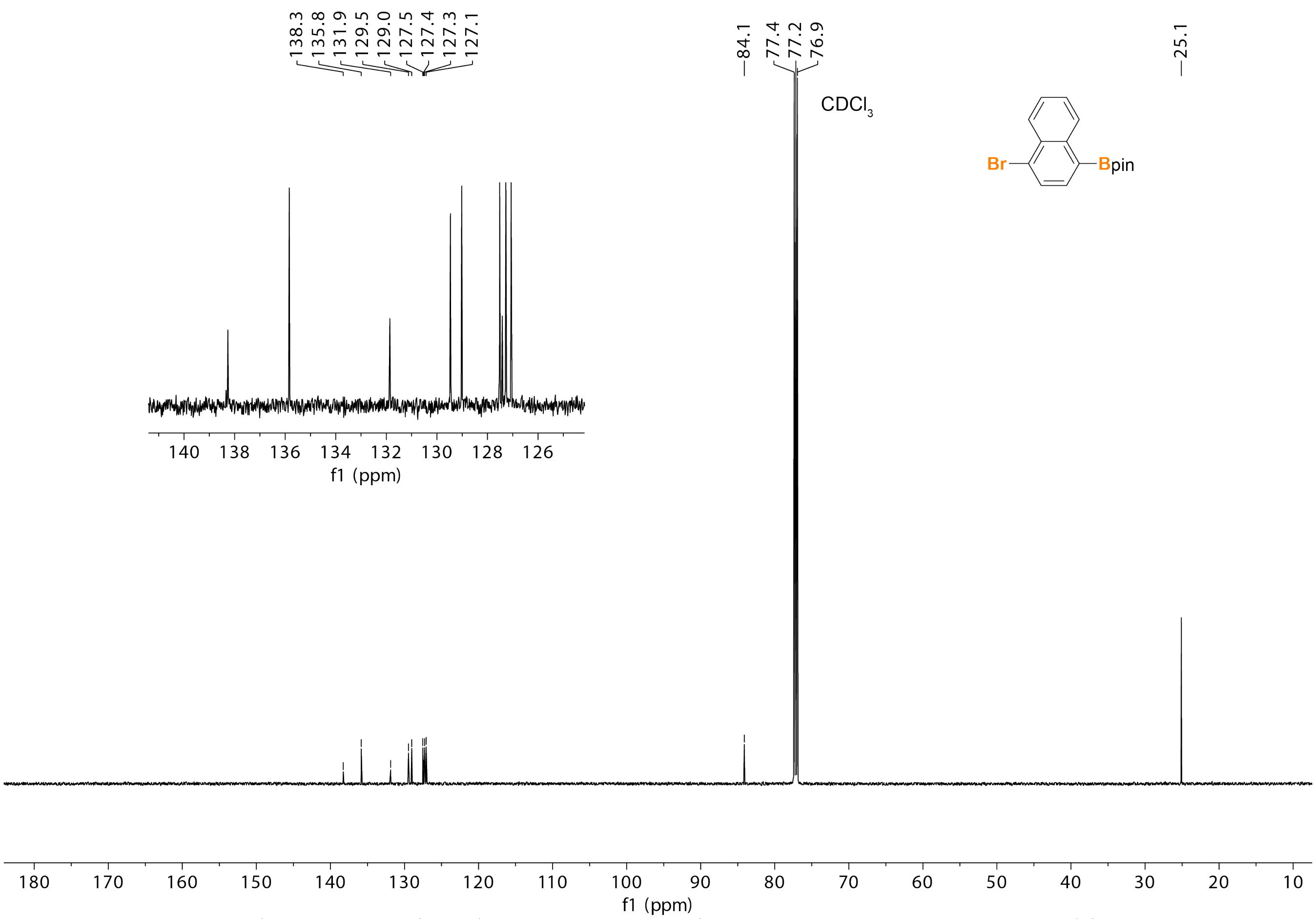

Figure S10. $^{13}C\{^{1}H\}$ NMR (151 MHz, $CDCl_3$) of 2-(4-bromonaphthalen-1-yl)-4,4,5,5-tetramethyl-1,3,2-dioxaborolane (**4**) at 24 °C.

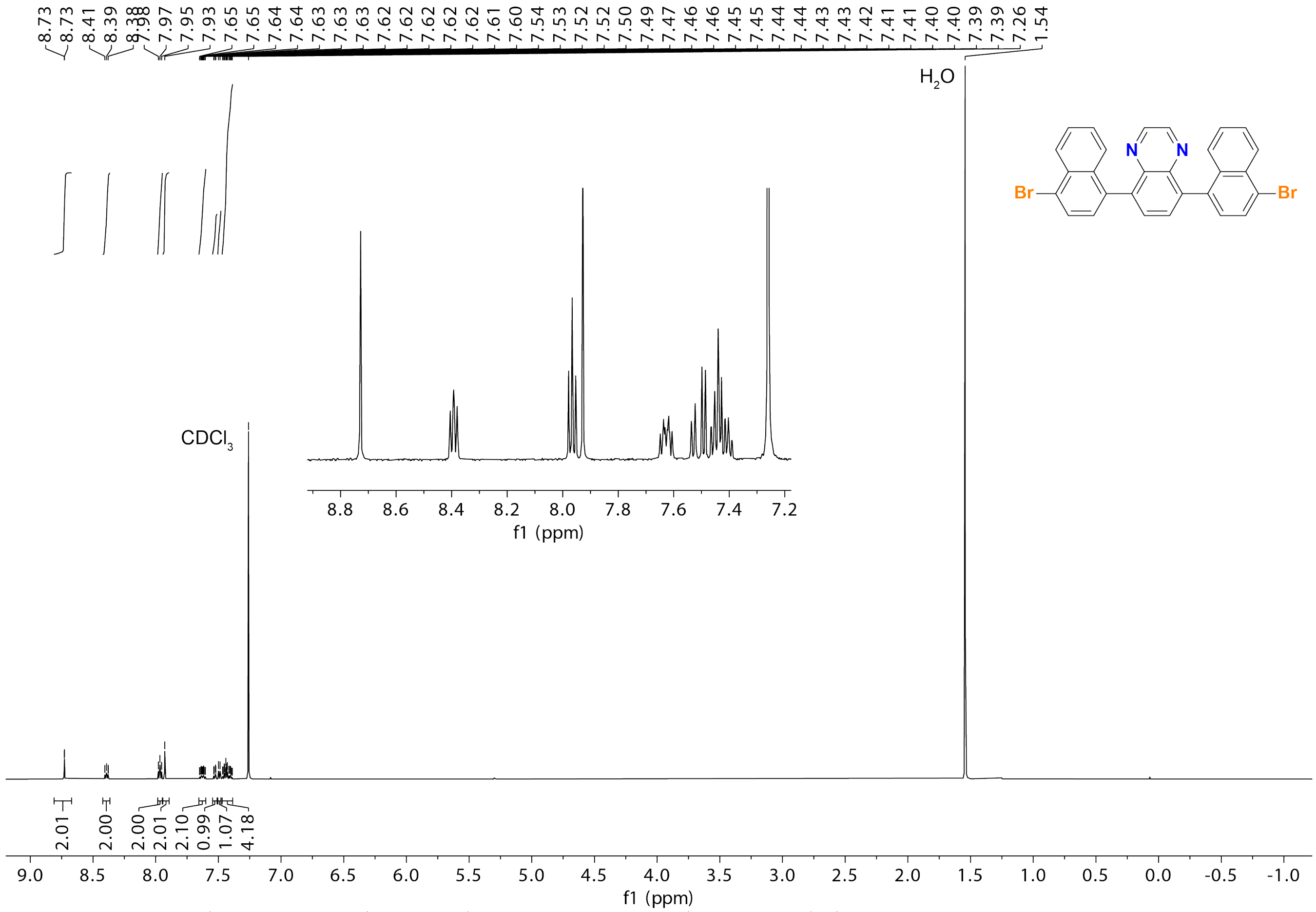

Figure S11. ^{1}H NMR (600 MHz, $CDCl_3$) of 5,8-bis(4-bromonaphthalen-1-yl)quinoxaline (**1a**) at 24 °C.

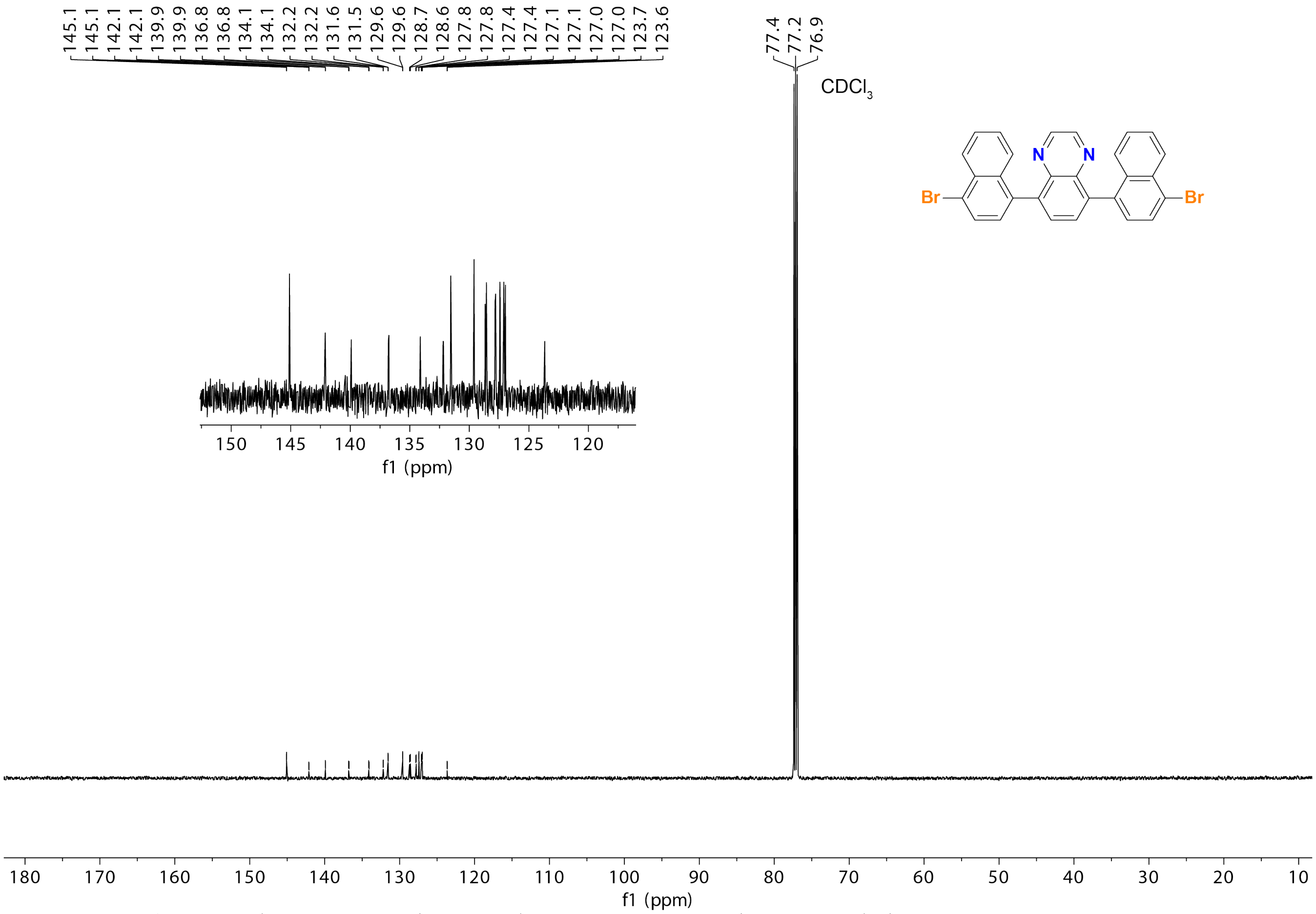

Figure S12. $^{13}C\{^1H\}$ NMR (151 MHz, $CDCl_3$) of 5,8-bis(4-bromonaphthalen-1-yl)quinoxaline (**1a**) at 24 °C.

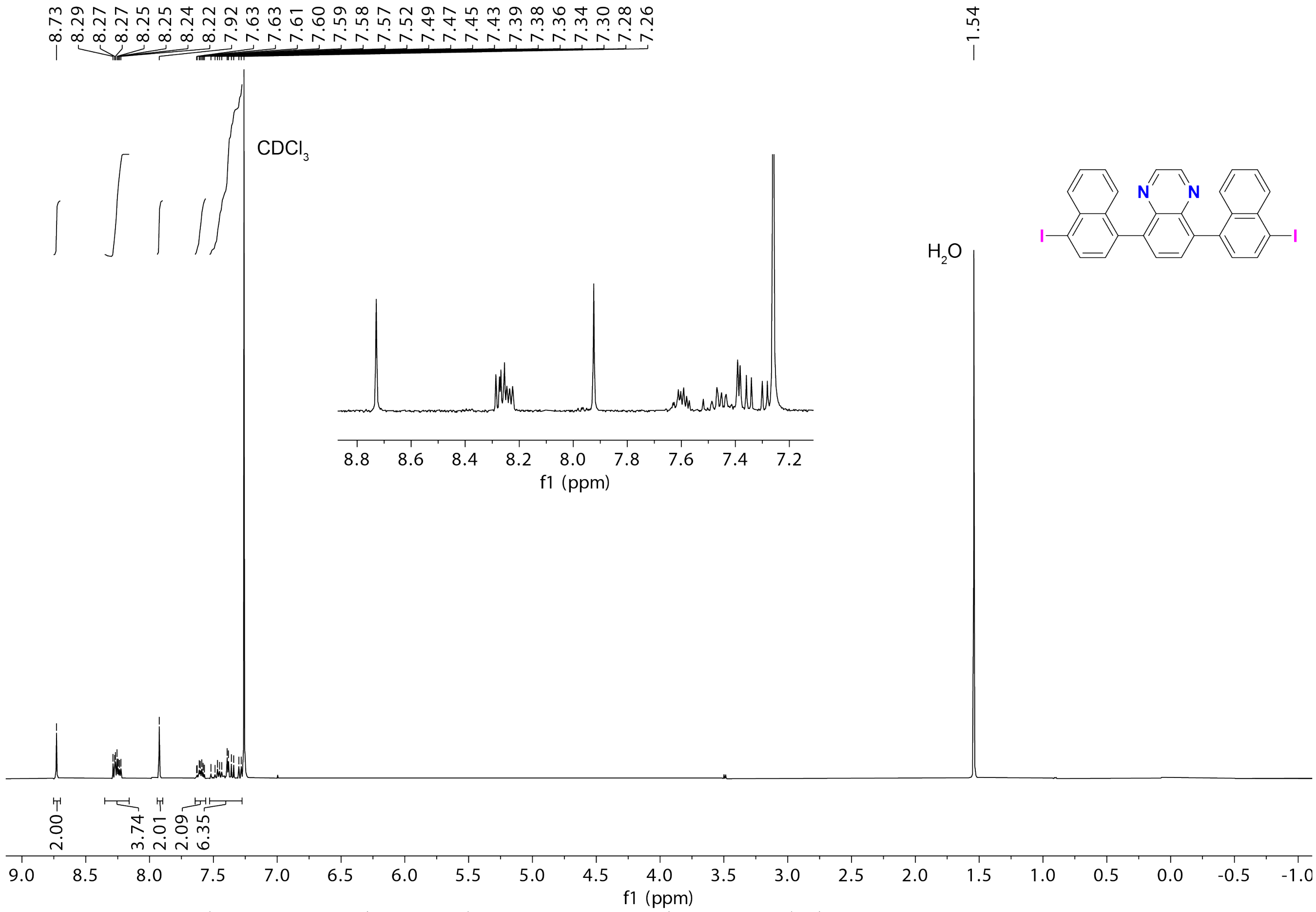

Figure S13. ^{1}H NMR (600 MHz, $CDCl_3$) of 5,8-bis(4-iodonaphthalen-1-yl)quinoxaline (**1b**) at 24 °C.

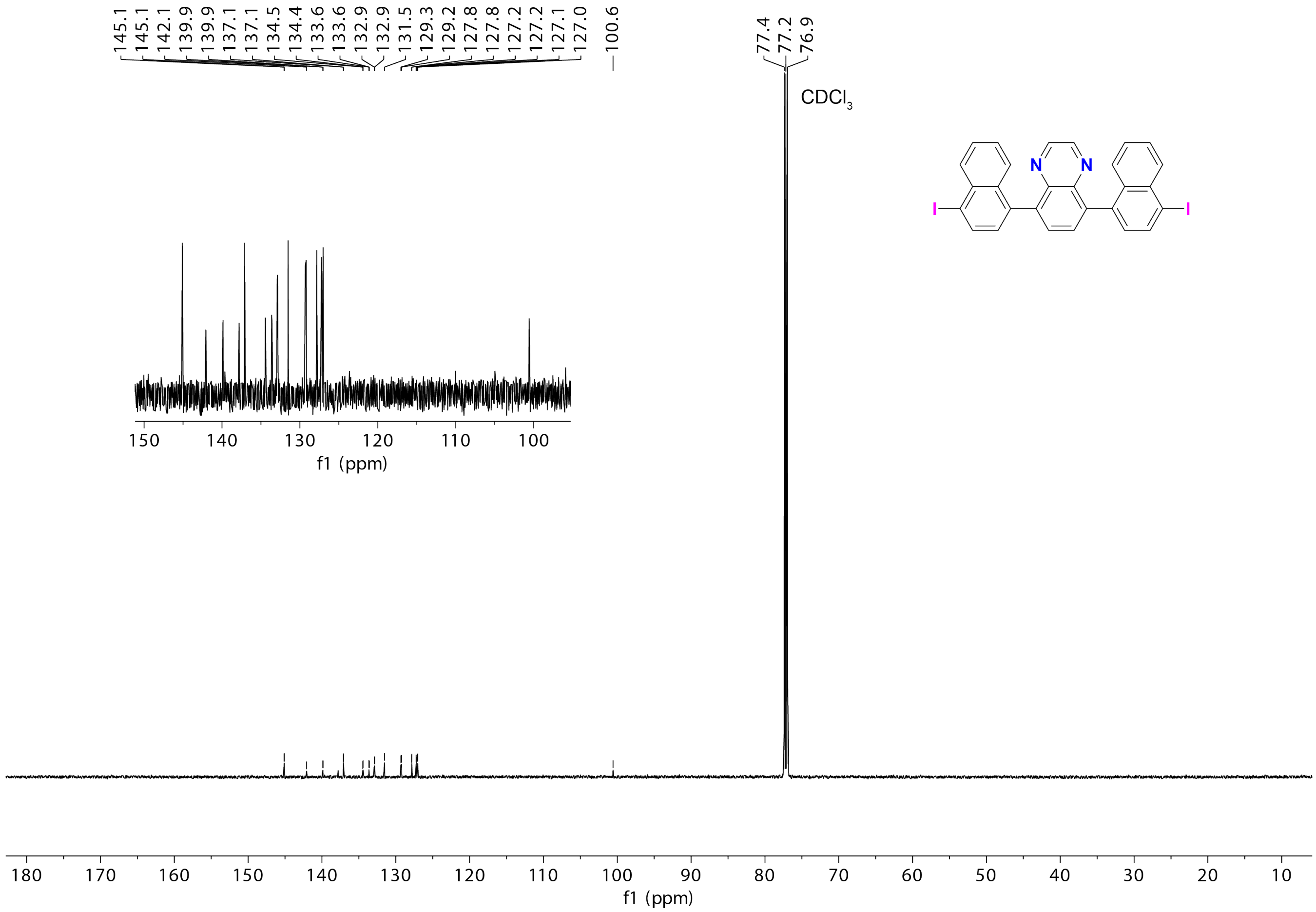

Figure S14. $^{13}C\{^{1}H\}$ NMR (151 MHz, $CDCl_3$) of 5,8-bis(4-iodonaphthalen-1-yl)quinoxaline (**1b**) at 24 °C.

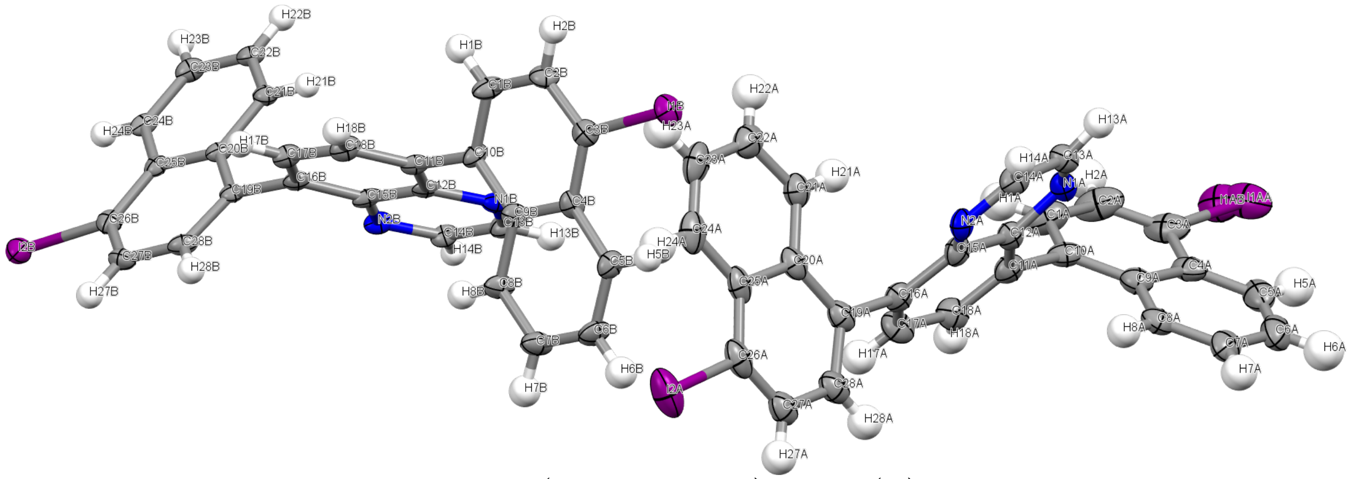

Figure S15. Single crystal X-ray structure diagram of 5,8-bis(4-iodonaphthalen-1-yl)quinoxaline (**1b**). ORTEP thermal ellipsoids are at the 50% probability level. Color coding: C (gray), N (blue), I (purple), H (white)

Table S1. Crystal data and structure refinement for 5,8-bis(4-iodonaphthalen-1-yl)quinoxaline (**1b**).

Identification code	CCDC 2237207	
Empirical formula	C28 H16 I2 N2	
Formula weight	634.23	
Temperature	100(2) K	
Wavelength	1.54184 Å	
Crystal system	Triclinic	
Space group	P –1	
Unit cell dimensions	*a* = 7.6031(2) Å	*α*= 92.127(2)°.
	b = 10.6541(2) Å	*β* = 93.111(2)°.
	c = 27.9954(6) Å	*γ* = 102.961(2)°.
Volume	2203.88(9) $Å^3$	
Z	4	
Density (calculated)	1.911 Mg/m^3	
Absorption coefficient	22.555 mm^{-1}	
F(000)	1216	
Crystal size	0.100 × 0.050 × 0.020 mm^3	
Theta range for data collection	3.166 to 68.248°.	
Index ranges	$-9 \le h \le 9$, $-12 \le k \le 12$, $-33 \le l \le 33$	
Reflections collected	41920	
Independent reflections	8061 [R(int) = 0.0820]	
Completeness to theta = 68.000°	100.0 %	
Absorption correction	Semi–empirical from equivalents	
Max. and min. transmission	1.00000 and 0.57099	
Refinement method	Full–matrix least–squares on F^2	
Data / restraints / parameters	8061 / 0 / 587	
Goodness–of–fit on F^2	1.083	
Final R indices [I>2sigma(I)]	R1 = 0.0607, wR2 = 0.1572	
R indices (all data)	R1 = 0.0676, wR2 = 0.1621	
Extinction coefficient	n/a	
Largest diff. peak and hole	2.015 and –1.961 e.$Å^{-3}$	

Table S2. Atomic coordinates (× 10^4) and equivalent isotropic displacement parameters (Å^2 × 10^3) for 5,8-bis(4-iodonaphthalen-1-yl)quinoxaline (**1b**). U(eq) is defined as one third of the trace of the orthogonalized U^{ij} tensor.

x	y	z	U(eq)		
I(1AA)	6081(11)	11048(5)	6814(4)	61(2)	
I(1AB)	5793(9)	11269(13)	6647(8)	57(2)	
I(2A)	10705(1)	3357(1)	2374(1)	58(1)	
N(1A)	7016(9)	5761(6)	5419(2)	29(1)	
N(2A)	7974(8)	4372(6)	4632(2)	27(1)	
C(1A)	5955(12)	9042(9)	5406(4)	46(2)	
C(2A)	5590(13)	9809(9)	5799(5)	53(3)	
C(3A)	6716(13)	10028(9)	6200(4)	51(3)	
C(4A)	8318(11)	9528(8)	6242(3)	36(2)	
C(5A)	9520(14)	9752(9)	6647(3)	48(2)	
C(6A)	10990(15)	9241(10)	6663(3)	50(2)	
C(7A)	11350(13)	8471(8)	6278(3)	40(2)	
C(8A)	10210(11)	8235(8)	5880(3)	34(2)	
C(9A)	8640(11)	8727(8)	5844(3)	32(2)	
C(10A)	7421(10)	8503(7)	5428(3)	33(2)	
C(11A)	7772(10)	7716(8)	5003(3)	30(2)	
C(12A)	7604(10)	6367(7)	5013(3)	27(2)	
C(13A)	6936(10)	4513(7)	5419(3)	29(2)	
C(14A)	7486(10)	3845(7)	5029(3)	29(2)	
C(15A)	7971(10)	5649(7)	4610(3)	28(2)	
C(16A)	8416(10)	6270(8)	4175(3)	30(2)	
C(17A)	8515(12)	7590(8)	4172(3)	37(2)	
C(18A)	8229(11)	8295(8)	4580(3)	36(2)	
C(19A)	8836(11)	5574(8)	3739(3)	30(2)	
C(20A)	7529(11)	4556(8)	3490(3)	31(2)	
C(21A)	5726(12)	4199(8)	3631(3)	34(2)	
C(22A)	4466(12)	3244(8)	3370(3)	40(2)	
C(23A)	4972(14)	2634(9)	2967(3)	44(2)	
C(24A)	6693(14)	2948(8)	2826(3)	41(2)	
C(25A)	8006(12)	3927(8)	3071(3)	34(2)	
C(26A)	9846(13)	4311(9)	2942(3)	40(2)	
C(27A)	11044(12)	5324(9)	3179(3)	36(2)	
C(28A)	10548(12)	5953(9)	3575(3)	37(2)	
I(1B)	6584(1)	−3423(1)	2608(1)	30(1)	
I(2B)	517(1)	4675(1)	−1447(1)	28(1)	
N(1B)	4689(7)	2028(5)	1576(2)	20(1)	
N(2B)	3460(7)	3478(5)	856(2)	20(1)	
C(1B)	3491(10)	−1687(7)	1581(3)	26(2)	
C(2B)	4075(10)	−2469(6)	1925(3)	24(1)	
C(3B)	5853(10)	−2206(7)	2093(3)	23(1)	
C(4B)	7164(9)	−1195(7)	1917(2)	21(1)	
C(5B)	9026(9)	−948(7)	2057(3)	24(1)	
C(6B)	10253(10)	24(8)	1868(3)	31(2)	
C(7B)	9653(10)	782(8)	1516(3)	31(2)	
C(8B)	7858(9)	575(7)	1375(3)	25(2)	
C(9B)	6555(9)	−410(6)	1564(2)	20(1)	
C(10B)	4684(9)	−659(6)	1413(2)	20(1)	
C(11B)	3995(8)	124(6)	1046(2)	17(1)	
C(12B)	4030(8)	1463(6)	1135(2)	18(1)	
C(13B)	4729(9)	3264(7)	1643(3)	25(2)	
C(14B)	4108(9)	3976(6)	1276(2)	20(1)	
C(15B)	3383(8)	2188(6)	772(3)	19(1)	
C(16B)	2643(8)	1569(6)	325(2)	19(1)	
C(17B)	2589(9)	276(6)	251(3)	21(1)	
C(18B)	3261(9)	−427(7)	608(3)	24(2)	
C(19B)	2040(8)	2301(6)	−79(2)	16(1)	
C(20B)	487(8)	2829(6)	−62(2)	17(1)	
C(21B)	−608(9)	2682(6)	339(2)	18(1)	
C(22B)	−2111(9)	3183(6)	343(3)	21(1)	

C(23B)	–2606(9)	3872(6)	–41(3)	22(1)
C(24B)	–1578(9)	4035(6)	–433(3)	20(1)
C(25B)	–28(8)	3508(6)	–459(2)	17(1)
C(26B)	1061(9)	3626(6)	–858(3)	23(1)
C(27B)	2517(9)	3068(6)	–874(3)	22(1)
C(28B)	2997(9)	2399(6)	–487(3)	22(1)

Table S3. Bond lengths [Å] and angles [°] for 5,8-bis(4-iodonaphthalen-1-yl)quinoxaline (**1b**).

I(1AA)–C(3A)	2.133(10)
I(1AB)–C(3A)	2.047(11)
I(2A)–C(26A)	2.069(9)
N(1A)–C(13A)	1.317(10)
N(1A)–C(12A)	1.374(10)
N(2A)–C(14A)	1.298(10)
N(2A)–C(15A)	1.364(10)
C(1A)–C(10A)	1.363(12)
C(1A)–C(2A)	1.423(14)
C(1A)–H(1A)	0.9500
C(2A)–C(3A)	1.355(16)
C(2A)–H(2A)	0.9500
C(3A)–C(4A)	1.436(14)
C(4A)–C(5A)	1.395(14)
C(4A)–C(9A)	1.442(12)
C(5A)–C(6A)	1.349(16)
C(5A)–H(5A)	0.9500
C(6A)–C(7A)	1.407(14)
C(6A)–H(6A)	0.9500
C(7A)–C(8A)	1.354(12)
C(7A)–H(7A)	0.9500
C(8A)–C(9A)	1.408(12)
C(8A)–H(8A)	0.9500
C(9A)–C(10A)	1.426(12)
C(10A)–C(11A)	1.501(12)
C(11A)–C(18A)	1.379(12)
C(11A)–C(12A)	1.416(11)
C(12A)–C(15A)	1.415(11)
C(13A)–C(14A)	1.415(11)
C(13A)–H(13A)	0.9500
C(14A)–H(14A)	0.9500
C(15A)–C(16A)	1.429(11)
C(16A)–C(17A)	1.391(11)
C(16A)–C(19A)	1.491(11)
C(17A)–C(18A)	1.398(13)
C(17A)–H(17A)	0.9500
C(18A)–H(18A)	0.9500
C(19A)–C(28A)	1.382(12)
C(19A)–C(20A)	1.426(12)
C(20A)–C(21A)	1.419(12)
C(20A)–C(25A)	1.433(11)
C(21A)–C(22A)	1.385(12)
C(21A)–H(21A)	0.9500
C(22A)–C(23A)	1.395(14)
C(22A)–H(22A)	0.9500
C(23A)–C(24A)	1.360(15)
C(23A)–H(23A)	0.9500
C(24A)–C(25A)	1.401(13)
C(24A)–H(24A)	0.9500
C(25A)–C(26A)	1.437(13)
C(26A)–C(27A)	1.369(13)
C(27A)–C(28A)	1.388(13)
C(27A)–H(27A)	0.9500
C(28A)–H(28A)	0.9500
I(1B)–C(3B)	2.109(7)
I(2B)–C(26B)	2.099(7)
N(1B)–C(13B)	1.317(9)
N(1B)–C(12B)	1.370(9)
N(2B)–C(14B)	1.296(9)
N(2B)–C(15B)	1.373(8)
C(1B)–C(10B)	1.373(10)
C(1B)–C(2B)	1.412(10)

C(1B)–H(1B)	0.9500
C(2B)–C(3B)	1.370(10)
C(2B)–H(2B)	0.9500
C(3B)–C(4B)	1.417(10)
C(4B)–C(5B)	1.412(10)
C(4B)–C(9B)	1.441(9)
C(5B)–C(6B)	1.372(11)
C(5B)–H(5B)	0.9500
C(6B)–C(7B)	1.417(11)
C(6B)–H(6B)	0.9500
C(7B)–C(8B)	1.367(10)
C(7B)–H(7B)	0.9500
C(8B)–C(9B)	1.412(10)
C(8B)–H(8B)	0.9500
C(9B)–C(10B)	1.425(9)
C(10B)–C(11B)	1.492(9)
C(11B)–C(18B)	1.374(10)
C(11B)–C(12B)	1.433(9)
C(12B)–C(15B)	1.432(9)
C(13B)–C(14B)	1.420(10)
C(13B)–H(13B)	0.9500
C(14B)–H(14B)	0.9500
C(15B)–C(16B)	1.419(10)
C(16B)–C(17B)	1.376(9)
C(16B)–C(19B)	1.505(9)
C(17B)–C(18B)	1.412(10)
C(17B)–H(17B)	0.9500
C(18B)–H(18B)	0.9500
C(19B)–C(28B)	1.382(10)
C(19B)–C(20B)	1.420(9)
C(20B)–C(21B)	1.425(9)
C(20B)–C(25B)	1.433(9)
C(21B)–C(22B)	1.366(9)
C(21B)–H(21B)	0.9500
C(22B)–C(23B)	1.407(10)
C(22B)–H(22B)	0.9500
C(23B)–C(24B)	1.374(10)
C(23B)–H(23B)	0.9500
C(24B)–C(25B)	1.419(9)
C(24B)–H(24B)	0.9500
C(25B)–C(26B)	1.419(10)
C(26B)–C(27B)	1.371(10)
C(27B)–C(28B)	1.394(10)
C(27B)–H(27B)	0.9500
C(28B)–H(28B)	0.9500
C(13A)–N(1A)–C(12A)	116.4(7)
C(14A)–N(2A)–C(15A)	116.1(7)
C(10A)–C(1A)–C(2A)	120.8(10)
C(10A)–C(1A)–H(1A)	119.6
C(2A)–C(1A)–H(1A)	119.6
C(3A)–C(2A)–C(1A)	120.1(9)
C(3A)–C(2A)–H(2A)	119.9
C(1A)–C(2A)–H(2A)	119.9
C(2A)–C(3A)–C(4A)	122.0(9)
C(2A)–C(3A)–I(1AB)	107.0(9)
C(4A)–C(3A)–I(1AB)	130.7(9)
C(2A)–C(3A)–I(1AA)	120.7(8)
C(4A)–C(3A)–I(1AA)	117.2(8)
C(5A)–C(4A)–C(3A)	123.4(9)
C(5A)–C(4A)–C(9A)	119.6(9)
C(3A)–C(4A)–C(9A)	116.9(8)
C(6A)–C(5A)–C(4A)	120.2(9)
C(6A)–C(5A)–H(5A)	119.9

C(4A)–C(5A)–H(5A)	119.9
C(5A)–C(6A)–C(7A)	121.3(9)
C(5A)–C(6A)–H(6A)	119.3
C(7A)–C(6A)–H(6A)	119.3
C(8A)–C(7A)–C(6A)	119.9(9)
C(8A)–C(7A)–H(7A)	120.0
C(6A)–C(7A)–H(7A)	120.0
C(7A)–C(8A)–C(9A)	121.3(9)
C(7A)–C(8A)–H(8A)	119.3
C(9A)–C(8A)–H(8A)	119.3
C(8A)–C(9A)–C(10A)	122.6(8)
C(8A)–C(9A)–C(4A)	117.5(8)
C(10A)–C(9A)–C(4A)	119.8(8)
C(1A)–C(10A)–C(9A)	120.2(8)
C(1A)–C(10A)–C(11A)	119.1(8)
C(9A)–C(10A)–C(11A)	120.6(7)
C(18A)–C(11A)–C(12A)	117.7(8)
C(18A)–C(11A)–C(10A)	120.2(7)
C(12A)–C(11A)–C(10A)	122.1(7)
N(1A)–C(12A)–C(15A)	120.6(7)
N(1A)–C(12A)–C(11A)	118.4(7)
C(15A)–C(12A)–C(11A)	121.0(7)
N(1A)–C(13A)–C(14A)	121.7(7)
N(1A)–C(13A)–H(13A)	119.2
C(14A)–C(13A)–H(13A)	119.2
N(2A)–C(14A)–C(13A)	123.6(7)
N(2A)–C(14A)–H(14A)	118.2
C(13A)–C(14A)–H(14A)	118.2
N(2A)–C(15A)–C(12A)	121.2(7)
N(2A)–C(15A)–C(16A)	118.7(7)
C(12A)–C(15A)–C(16A)	120.0(7)
C(17A)–C(16A)–C(15A)	117.5(7)
C(17A)–C(16A)–C(19A)	119.8(7)
C(15A)–C(16A)–C(19A)	122.7(7)
C(16A)–C(17A)–C(18A)	121.7(8)
C(16A)–C(17A)–H(17A)	119.1
C(18A)–C(17A)–H(17A)	119.1
C(11A)–C(18A)–C(17A)	122.0(8)
C(11A)–C(18A)–H(18A)	119.0
C(17A)–C(18A)–H(18A)	119.0
C(28A)–C(19A)–C(20A)	120.1(8)
C(28A)–C(19A)–C(16A)	117.8(8)
C(20A)–C(19A)–C(16A)	122.0(7)
C(21A)–C(20A)–C(19A)	121.5(7)
C(21A)–C(20A)–C(25A)	118.8(8)
C(19A)–C(20A)–C(25A)	119.6(8)
C(22A)–C(21A)–C(20A)	120.2(8)
C(22A)–C(21A)–H(21A)	119.9
C(20A)–C(21A)–H(21A)	119.9
C(21A)–C(22A)–C(23A)	119.8(9)
C(21A)–C(22A)–H(22A)	120.1
C(23A)–C(22A)–H(22A)	120.1
C(24A)–C(23A)–C(22A)	121.3(9)
C(24A)–C(23A)–H(23A)	119.3
C(22A)–C(23A)–H(23A)	119.3
C(23A)–C(24A)–C(25A)	121.0(9)
C(23A)–C(24A)–H(24A)	119.5
C(25A)–C(24A)–H(24A)	119.5
C(24A)–C(25A)–C(20A)	118.8(8)
C(24A)–C(25A)–C(26A)	124.1(8)
C(20A)–C(25A)–C(26A)	117.1(8)
C(27A)–C(26A)–C(25A)	121.5(8)
C(27A)–C(26A)–I(2A)	118.8(7)
C(25A)–C(26A)–I(2A)	119.7(7)

C(26A)–C(27A)–C(28A)	120.6(8)
C(26A)–C(27A)–H(27A)	119.7
C(28A)–C(27A)–H(27A)	119.7
C(19A)–C(28A)–C(27A)	120.8(9)
C(19A)–C(28A)–H(28A)	119.6
C(27A)–C(28A)–H(28A)	119.6
C(13B)–N(1B)–C(12B)	116.8(6)
C(14B)–N(2B)–C(15B)	116.7(6)
C(10B)–C(1B)–C(2B)	120.8(6)
C(10B)–C(1B)–H(1B)	119.6
C(2B)–C(1B)–H(1B)	119.6
C(3B)–C(2B)–C(1B)	120.0(6)
C(3B)–C(2B)–H(2B)	120.0
C(1B)–C(2B)–H(2B)	120.0
C(2B)–C(3B)–C(4B)	121.8(7)
C(2B)–C(3B)–I(1B)	117.0(5)
C(4B)–C(3B)–I(1B)	121.1(5)
C(5B)–C(4B)–C(3B)	123.6(6)
C(5B)–C(4B)–C(9B)	118.7(6)
C(3B)–C(4B)–C(9B)	117.7(6)
C(6B)–C(5B)–C(4B)	121.6(7)
C(6B)–C(5B)–H(5B)	119.2
C(4B)–C(5B)–H(5B)	119.2
C(5B)–C(6B)–C(7B)	119.5(7)
C(5B)–C(6B)–H(6B)	120.2
C(7B)–C(6B)–H(6B)	120.2
C(8B)–C(7B)–C(6B)	120.4(7)
C(8B)–C(7B)–H(7B)	119.8
C(6B)–C(7B)–H(7B)	119.8
C(7B)–C(8B)–C(9B)	121.5(7)
C(7B)–C(8B)–H(8B)	119.2
C(9B)–C(8B)–H(8B)	119.2
C(8B)–C(9B)–C(10B)	122.4(6)
C(8B)–C(9B)–C(4B)	118.2(6)
C(10B)–C(9B)–C(4B)	119.5(6)
C(1B)–C(10B)–C(9B)	120.1(6)
C(1B)–C(10B)–C(11B)	118.5(6)
C(9B)–C(10B)–C(11B)	121.3(6)
C(18B)–C(11B)–C(12B)	117.1(6)
C(18B)–C(11B)–C(10B)	120.7(6)
C(12B)–C(11B)–C(10B)	122.2(6)
N(1B)–C(12B)–C(15B)	121.0(6)
N(1B)–C(12B)–C(11B)	118.5(6)
C(15B)–C(12B)–C(11B)	120.6(6)
N(1B)–C(13B)–C(14B)	121.5(7)
N(1B)–C(13B)–H(13B)	119.2
C(14B)–C(13B)–H(13B)	119.2
N(2B)–C(14B)–C(13B)	123.7(6)
N(2B)–C(14B)–H(14B)	118.2
C(13B)–C(14B)–H(14B)	118.2
N(2B)–C(15B)–C(16B)	120.0(6)
N(2B)–C(15B)–C(12B)	120.2(6)
C(16B)–C(15B)–C(12B)	119.8(6)
C(17B)–C(16B)–C(15B)	118.6(6)
C(17B)–C(16B)–C(19B)	119.3(6)
C(15B)–C(16B)–C(19B)	121.9(6)
C(16B)–C(17B)–C(18B)	121.2(7)
C(16B)–C(17B)–H(17B)	119.4
C(18B)–C(17B)–H(17B)	119.4
C(11B)–C(18B)–C(17B)	122.6(6)
C(11B)–C(18B)–H(18B)	118.7
C(17B)–C(18B)–H(18B)	118.7
C(28B)–C(19B)–C(20B)	119.6(6)
C(28B)–C(19B)–C(16B)	118.0(6)

C(20B)–C(19B)–C(16B)	122.3(6)
C(19B)–C(20B)–C(21B)	121.7(6)
C(19B)–C(20B)–C(25B)	119.7(6)
C(21B)–C(20B)–C(25B)	118.6(6)
C(22B)–C(21B)–C(20B)	120.6(6)
C(22B)–C(21B)–H(21B)	119.7
C(20B)–C(21B)–H(21B)	119.7
C(21B)–C(22B)–C(23B)	121.2(6)
C(21B)–C(22B)–H(22B)	119.4
C(23B)–C(22B)–H(22B)	119.4
C(24B)–C(23B)–C(22B)	119.8(6)
C(24B)–C(23B)–H(23B)	120.1
C(22B)–C(23B)–H(23B)	120.1
C(23B)–C(24B)–C(25B)	121.1(6)
C(23B)–C(24B)–H(24B)	119.5
C(25B)–C(24B)–H(24B)	119.5
C(24B)–C(25B)–C(26B)	123.4(6)
C(24B)–C(25B)–C(20B)	118.8(6)
C(26B)–C(25B)–C(20B)	117.8(6)
C(27B)–C(26B)–C(25B)	121.4(6)
C(27B)–C(26B)–I(2B)	117.3(5)
C(25B)–C(26B)–I(2B)	121.3(5)
C(26B)–C(27B)–C(28B)	120.4(6)
C(26B)–C(27B)–H(27B)	119.8
C(28B)–C(27B)–H(27B)	119.8
C(19B)–C(28B)–C(27B)	121.0(6)
C(19B)–C(28B)–H(28B)	119.5
C(27B)–C(28B)–H(28B)	119.5

Symmetry transformations used to generate equivalent atoms:

Table S4. Anisotropic displacement parameters ($Å^2 \times 10^3$) for 5,8-bis(4-iodonaphthalen-1-yl)quinoxaline (**1b**). The anisotropic displacement factor exponent takes the form: $-2\pi^2[h^2a^{*2}U^{11} + ... + 2 h k a^* b^* U^{12}]$

	U^{11}	U^{22}	U^{33}	U^{23}	U^{13}	U^{12}
I(1AA)	70(2)	33(1)	77(3)	−21(1)	35(2)	1(1)
I(1AB)	56(2)	42(2)	70(4)	−12(3)	9(2)	8(1)
I(2A)	83(1)	73(1)	34(1)	12(1)	14(1)	48(1)
N(1A)	30(3)	37(4)	25(3)	3(3)	3(3)	15(3)
N(2A)	29(3)	32(3)	23(3)	3(3)	−4(2)	15(3)
C(1A)	35(5)	35(5)	71(7)	−3(4)	0(4)	13(4)
C(2A)	36(5)	29(4)	94(8)	−19(5)	9(5)	10(4)
C(3A)	46(5)	29(4)	72(7)	−17(4)	21(5)	−4(4)
C(4A)	33(4)	27(4)	44(5)	0(3)	12(4)	−3(3)
C(5A)	60(6)	35(5)	36(5)	−2(4)	5(4)	−12(4)
C(6A)	61(6)	46(5)	36(5)	13(4)	−2(4)	−1(5)
C(7A)	44(5)	36(5)	38(5)	16(4)	−2(4)	4(4)
C(8A)	37(4)	31(4)	36(4)	12(3)	10(3)	6(3)
C(9A)	32(4)	27(4)	38(4)	7(3)	7(3)	5(3)
C(10A)	26(4)	22(4)	51(5)	5(3)	6(3)	3(3)
C(11A)	26(4)	30(4)	36(4)	2(3)	−3(3)	10(3)
C(12A)	25(4)	30(4)	28(4)	4(3)	0(3)	12(3)
C(13A)	29(4)	28(4)	32(4)	7(3)	5(3)	11(3)
C(14A)	30(4)	22(4)	36(4)	5(3)	−2(3)	9(3)
C(15A)	30(4)	28(4)	28(4)	3(3)	−5(3)	10(3)
C(16A)	26(4)	34(4)	32(4)	5(3)	−2(3)	13(3)
C(17A)	43(5)	31(4)	40(5)	11(4)	8(4)	14(4)
C(18A)	37(4)	34(4)	41(5)	9(4)	3(4)	13(4)
C(19A)	35(4)	32(4)	28(4)	11(3)	5(3)	15(3)
C(20A)	42(4)	30(4)	27(4)	11(3)	4(3)	16(3)
C(21A)	41(5)	36(4)	28(4)	6(3)	−2(3)	15(4)
C(22A)	42(5)	36(4)	41(5)	14(4)	−8(4)	8(4)
C(23A)	59(6)	36(5)	36(5)	2(4)	−18(4)	17(4)
C(24A)	63(6)	34(4)	31(4)	7(3)	−9(4)	23(4)
C(25A)	47(5)	41(4)	22(4)	11(3)	0(3)	23(4)
C(26A)	54(5)	48(5)	27(4)	15(4)	6(4)	28(4)
C(27A)	39(5)	44(5)	31(4)	13(4)	4(3)	17(4)
C(28A)	39(5)	43(5)	34(4)	15(4)	5(3)	16(4)
I(1B)	38(1)	25(1)	30(1)	9(1)	3(1)	14(1)
I(2B)	28(1)	26(1)	27(1)	6(1)	−1(1)	3(1)
N(1B)	15(3)	19(3)	28(3)	6(2)	4(2)	5(2)
N(2B)	18(3)	11(3)	32(3)	3(2)	1(2)	5(2)
C(1B)	20(3)	15(3)	42(4)	5(3)	−2(3)	2(3)
C(2B)	25(4)	15(3)	32(4)	6(3)	4(3)	2(3)
C(3B)	27(4)	18(3)	25(3)	−1(3)	3(3)	8(3)
C(4B)	24(3)	23(3)	22(3)	3(3)	3(3)	15(3)
C(5B)	24(3)	20(3)	29(4)	3(3)	−4(3)	9(3)
C(6B)	17(3)	35(4)	41(4)	5(3)	−3(3)	7(3)
C(7B)	20(4)	32(4)	41(4)	13(3)	4(3)	6(3)
C(8B)	17(3)	25(4)	36(4)	10(3)	3(3)	10(3)
C(9B)	19(3)	18(3)	26(3)	2(3)	4(3)	10(3)
C(10B)	17(3)	16(3)	26(3)	1(3)	−4(3)	5(3)
C(11B)	11(3)	13(3)	30(4)	4(3)	2(2)	7(2)
C(12B)	8(3)	18(3)	30(4)	4(3)	3(2)	6(2)
C(13B)	23(3)	18(3)	31(4)	0(3)	−1(3)	2(3)
C(14B)	20(3)	11(3)	28(4)	−2(3)	1(3)	3(2)
C(15B)	11(3)	13(3)	34(4)	2(3)	−1(3)	4(2)
C(16B)	12(3)	17(3)	27(3)	2(3)	2(2)	5(2)
C(17B)	17(3)	16(3)	31(4)	1(3)	0(3)	6(3)
C(18B)	19(3)	19(3)	37(4)	2(3)	1(3)	10(3)
C(19B)	15(3)	10(3)	23(3)	2(2)	1(2)	0(2)
C(20B)	14(3)	11(3)	26(3)	2(2)	0(2)	3(2)

C(21B)	17(3)	15(3)	23(3)	3(2)	2(3)	2(2)
C(22B)	15(3)	17(3)	29(4)	1(3)	2(3)	1(3)
C(23B)	18(3)	15(3)	33(4)	0(3)	−1(3)	6(3)
C(24B)	17(3)	10(3)	32(4)	6(3)	−5(3)	3(2)
C(25B)	18(3)	11(3)	22(3)	−1(2)	−2(2)	1(2)
C(26B)	25(3)	15(3)	26(4)	2(3)	−1(3)	2(3)
C(27B)	21(3)	20(3)	25(3)	0(3)	8(3)	3(3)
C(28B)	15(3)	17(3)	35(4)	1(3)	3(3)	5(2)

Table S5. Hydrogen coordinates (× 10^4) and isotropic displacement parameters (Å^2 × 10^3) for 5,8-bis(4-iodonaphthalen-1-yl)quinoxaline (**1b**).

	x	y	z	U(eq)
H(1A)	5168	8904	5124	55
H(2A)	4554	10169	5780	63
H(5A)	9302	10267	6912	57
H(6A)	11799	9403	6941	60
H(7A)	12392	8118	6297	48
H(8A)	10475	7726	5619	41
H(13A)	6498	4050	5688	34
H(14A)	7499	2962	5059	34
H(17A)	8783	8023	3885	44
H(18A)	8353	9200	4567	43
H(21A)	5382	4616	3905	41
H(22A)	3260	3004	3465	48
H(23A)	4096	1987	2787	53
H(24A)	7009	2495	2556	49
H(27A)	12225	5600	3071	44
H(28A)	11396	6652	3737	45
H(1B)	2257	−1875	1466	31
H(2B)	3237	−3177	2039	29
H(5B)	9439	−1467	2287	29
H(6B)	11497	187	1973	37
H(7B)	10503	1439	1378	37
H(8B)	7478	1106	1143	30
H(13B)	5183	3690	1943	30
H(14B)	4171	4866	1342	24
H(17B)	2090	−151	−46	26
H(18B)	3204	−1318	543	29
H(21B)	−289	2232	605	22
H(22B)	−2836	3064	611	25
H(23B)	−3647	4224	−31	26
H(24B)	−1911	4509	−690	24
H(27B)	3202	3137	−1149	26
H(28B)	3997	2005	−504	27

Table S6. Torsion angles [°] for 5,8-bis(4-iodonaphthalen-1-yl)quinoxaline (**1b**).

C(10A)–C(1A)–C(2A)–C(3A)	0.9(15)
C(1A)–C(2A)–C(3A)–C(4A)	1.3(15)
C(1A)–C(2A)–C(3A)–I(1AB)	176.0(10)
C(1A)–C(2A)–C(3A)–I(1AA)	–175.1(8)
C(2A)–C(3A)–C(4A)–C(5A)	178.9(9)
I(1AB)–C(3A)–C(4A)–C(5A)	5.7(16)
I(1AA)–C(3A)–C(4A)–C(5A)	–4.6(12)
C(2A)–C(3A)–C(4A)–C(9A)	–2.7(13)
I(1AB)–C(3A)–C(4A)–C(9A)	–175.9(10)
I(1AA)–C(3A)–C(4A)–C(9A)	173.8(6)
C(3A)–C(4A)–C(5A)–C(6A)	179.4(9)
C(9A)–C(4A)–C(5A)–C(6A)	1.0(13)
C(4A)–C(5A)–C(6A)–C(7A)	–0.1(14)
C(5A)–C(6A)–C(7A)–C(8A)	0.1(14)
C(6A)–C(7A)–C(8A)–C(9A)	–1.1(13)
C(7A)–C(8A)–C(9A)–C(10A)	179.5(8)
C(7A)–C(8A)–C(9A)–C(4A)	1.9(12)
C(5A)–C(4A)–C(9A)–C(8A)	–1.9(12)
C(3A)–C(4A)–C(9A)–C(8A)	179.7(8)
C(5A)–C(4A)–C(9A)–C(10A)	–179.5(8)
C(3A)–C(4A)–C(9A)–C(10A)	2.0(11)
C(2A)–C(1A)–C(10A)–C(9A)	–1.5(14)
C(2A)–C(1A)–C(10A)–C(11A)	–179.1(8)
C(8A)–C(9A)–C(10A)–C(1A)	–177.6(8)
C(4A)–C(9A)–C(10A)–C(1A)	0.0(12)
C(8A)–C(9A)–C(10A)–C(11A)	0.0(12)
C(4A)–C(9A)–C(10A)–C(11A)	177.6(7)
C(1A)–C(10A)–C(11A)–C(18A)	66.4(11)
C(9A)–C(10A)–C(11A)–C(18A)	–111.2(9)
C(1A)–C(10A)–C(11A)–C(12A)	–111.9(9)
C(9A)–C(10A)–C(11A)–C(12A)	70.5(10)
C(13A)–N(1A)–C(12A)–C(15A)	4.3(10)
C(13A)–N(1A)–C(12A)–C(11A)	–177.8(7)
C(18A)–C(11A)–C(12A)–N(1A)	–175.1(7)
C(10A)–C(11A)–C(12A)–N(1A)	3.2(11)
C(18A)–C(11A)–C(12A)–C(15A)	2.7(11)
C(10A)–C(11A)–C(12A)–C(15A)	–179.0(7)
C(12A)–N(1A)–C(13A)–C(14A)	2.1(11)
C(15A)–N(2A)–C(14A)–C(13A)	1.4(11)
N(1A)–C(13A)–C(14A)–N(2A)	–5.3(12)
C(14A)–N(2A)–C(15A)–C(12A)	5.2(10)
C(14A)–N(2A)–C(15A)–C(16A)	–177.2(7)
N(1A)–C(12A)–C(15A)–N(2A)	–8.4(11)
C(11A)–C(12A)–C(15A)–N(2A)	173.9(7)
N(1A)–C(12A)–C(15A)–C(16A)	174.1(7)
C(11A)–C(12A)–C(15A)–C(16A)	–3.7(11)
N(2A)–C(15A)–C(16A)–C(17A)	–176.0(7)
C(12A)–C(15A)–C(16A)–C(17A)	1.6(11)
N(2A)–C(15A)–C(16A)–C(19A)	1.7(11)
C(12A)–C(15A)–C(16A)–C(19A)	179.3(7)
C(15A)–C(16A)–C(17A)–C(18A)	1.3(12)
C(19A)–C(16A)–C(17A)–C(18A)	–176.5(8)
C(12A)–C(11A)–C(18A)–C(17A)	0.2(12)
C(10A)–C(11A)–C(18A)–C(17A)	–178.1(8)
C(16A)–C(17A)–C(18A)–C(11A)	–2.3(13)
C(17A)–C(16A)–C(19A)–C(28A)	60.7(10)
C(15A)–C(16A)–C(19A)–C(28A)	–117.0(9)
C(17A)–C(16A)–C(19A)–C(20A)	–118.7(9)
C(15A)–C(16A)–C(19A)–C(20A)	63.6(10)
C(28A)–C(19A)–C(20A)–C(21A)	–176.0(7)
C(16A)–C(19A)–C(20A)–C(21A)	3.4(11)
C(28A)–C(19A)–C(20A)–C(25A)	0.2(11)

C(16A)–C(19A)–C(20A)–C(25A)	179.6(7)
C(19A)–C(20A)–C(21A)–C(22A)	177.2(7)
C(25A)–C(20A)–C(21A)–C(22A)	1.0(11)
C(20A)–C(21A)–C(22A)–C(23A)	–0.2(12)
C(21A)–C(22A)–C(23A)–C(24A)	0.8(12)
C(22A)–C(23A)–C(24A)–C(25A)	–2.2(12)
C(23A)–C(24A)–C(25A)–C(20A)	3.0(12)
C(23A)–C(24A)–C(25A)–C(26A)	–179.9(8)
C(21A)–C(20A)–C(25A)–C(24A)	–2.4(11)
C(19A)–C(20A)–C(25A)–C(24A)	–178.7(7)
C(21A)–C(20A)–C(25A)–C(26A)	–179.7(7)
C(19A)–C(20A)–C(25A)–C(26A)	4.0(11)
C(24A)–C(25A)–C(26A)–C(27A)	176.3(8)
C(20A)–C(25A)–C(26A)–C(27A)	–6.6(11)
C(24A)–C(25A)–C(26A)–I(2A)	–1.8(10)
C(20A)–C(25A)–C(26A)–I(2A)	175.3(5)
C(25A)–C(26A)–C(27A)–C(28A)	4.8(12)
I(2A)–C(26A)–C(27A)–C(28A)	–177.1(6)
C(20A)–C(19A)–C(28A)–C(27A)	–2.3(12)
C(16A)–C(19A)–C(28A)–C(27A)	178.4(7)
C(26A)–C(27A)–C(28A)–C(19A)	–0.3(12)
C(10B)–C(1B)–C(2B)–C(3B)	–0.1(11)
C(1B)–C(2B)–C(3B)–C(4B)	–2.8(11)
C(1B)–C(2B)–C(3B)–I(1B)	179.6(5)
C(2B)–C(3B)–C(4B)–C(5B)	–175.6(7)
I(1B)–C(3B)–C(4B)–C(5B)	2.0(9)
C(2B)–C(3B)–C(4B)–C(9B)	2.2(10)
I(1B)–C(3B)–C(4B)–C(9B)	179.8(5)
C(3B)–C(4B)–C(5B)–C(6B)	178.4(7)
C(9B)–C(4B)–C(5B)–C(6B)	0.6(11)
C(4B)–C(5B)–C(6B)–C(7B)	–1.5(12)
C(5B)–C(6B)–C(7B)–C(8B)	1.9(13)
C(6B)–C(7B)–C(8B)–C(9B)	–1.4(12)
C(7B)–C(8B)–C(9B)–C(10B)	–178.6(7)
C(7B)–C(8B)–C(9B)–C(4B)	0.5(11)
C(5B)–C(4B)–C(9B)–C(8B)	–0.1(10)
C(3B)–C(4B)–C(9B)–C(8B)	–178.1(6)
C(5B)–C(4B)–C(9B)–C(10B)	179.0(6)
C(3B)–C(4B)–C(9B)–C(10B)	1.1(10)
C(2B)–C(1B)–C(10B)–C(9B)	3.4(11)
C(2B)–C(1B)–C(10B)–C(11B)	179.3(7)
C(8B)–C(9B)–C(10B)–C(1B)	175.2(7)
C(4B)–C(9B)–C(10B)–C(1B)	–3.9(10)
C(8B)–C(9B)–C(10B)–C(11B)	–0.5(10)
C(4B)–C(9B)–C(10B)–C(11B)	–179.7(6)
C(1B)–C(10B)–C(11B)–C(18B)	–61.2(9)
C(9B)–C(10B)–C(11B)–C(18B)	114.6(7)
C(1B)–C(10B)–C(11B)–C(12B)	117.5(7)
C(9B)–C(10B)–C(11B)–C(12B)	–66.6(9)
C(13B)–N(1B)–C(12B)–C(15B)	–1.4(9)
C(13B)–N(1B)–C(12B)–C(11B)	178.9(6)
C(18B)–C(11B)–C(12B)–N(1B)	177.5(6)
C(10B)–C(11B)–C(12B)–N(1B)	–1.3(9)
C(18B)–C(11B)–C(12B)–C(15B)	–2.2(9)
C(10B)–C(11B)–C(12B)–C(15B)	179.0(6)
C(12B)–N(1B)–C(13B)–C(14B)	0.4(10)
C(15B)–N(2B)–C(14B)–C(13B)	0.2(10)
N(1B)–C(13B)–C(14B)–N(2B)	0.2(11)
C(14B)–N(2B)–C(15B)–C(16B)	178.3(6)
C(14B)–N(2B)–C(15B)–C(12B)	–1.2(9)
N(1B)–C(12B)–C(15B)–N(2B)	1.8(9)
C(11B)–C(12B)–C(15B)–N(2B)	–178.5(6)
N(1B)–C(12B)–C(15B)–C(16B)	–177.6(6)
C(11B)–C(12B)–C(15B)–C(16B)	2.1(9)

N(2B)–C(15B)–C(16B)–C(17B)	179.8(6)
C(12B)–C(15B)–C(16B)–C(17B)	–0.8(9)
N(2B)–C(15B)–C(16B)–C(19B)	4.1(9)
C(12B)–C(15B)–C(16B)–C(19B)	–176.5(6)
C(15B)–C(16B)–C(17B)–C(18B)	–0.4(10)
C(19B)–C(16B)–C(17B)–C(18B)	175.5(6)
C(12B)–C(11B)–C(18B)–C(17B)	1.1(10)
C(10B)–C(11B)–C(18B)–C(17B)	179.9(6)
C(16B)–C(17B)–C(18B)–C(11B)	0.2(11)
C(17B)–C(16B)–C(19B)–C(28B)	–62.6(8)
C(15B)–C(16B)–C(19B)–C(28B)	113.1(7)
C(17B)–C(16B)–C(19B)–C(20B)	114.2(7)
C(15B)–C(16B)–C(19B)–C(20B)	–70.1(8)
C(28B)–C(19B)–C(20B)–C(21B)	176.1(6)
C(16B)–C(19B)–C(20B)–C(21B)	–0.7(9)
C(28B)–C(19B)–C(20B)–C(25B)	–2.8(9)
C(16B)–C(19B)–C(20B)–C(25B)	–179.5(6)
C(19B)–C(20B)–C(21B)–C(22B)	–179.0(6)
C(25B)–C(20B)–C(21B)–C(22B)	–0.2(9)
C(20B)–C(21B)–C(22B)–C(23B)	–1.0(10)
C(21B)–C(22B)–C(23B)–C(24B)	0.8(10)
C(22B)–C(23B)–C(24B)–C(25B)	0.6(10)
C(23B)–C(24B)–C(25B)–C(26B)	178.8(6)
C(23B)–C(24B)–C(25B)–C(20B)	–1.8(9)
C(19B)–C(20B)–C(25B)–C(24B)	–179.6(6)
C(21B)–C(20B)–C(25B)–C(24B)	1.6(9)
C(19B)–C(20B)–C(25B)–C(26B)	–0.1(9)
C(21B)–C(20B)–C(25B)–C(26B)	–179.0(6)
C(24B)–C(25B)–C(26B)–C(27B)	–178.1(6)
C(20B)–C(25B)–C(26B)–C(27B)	2.5(9)
C(24B)–C(25B)–C(26B)–I(2B)	2.4(9)
C(20B)–C(25B)–C(26B)–I(2B)	–177.0(4)
C(25B)–C(26B)–C(27B)–C(28B)	–2.0(10)
I(2B)–C(26B)–C(27B)–C(28B)	177.5(5)
C(20B)–C(19B)–C(28B)–C(27B)	3.4(9)
C(16B)–C(19B)–C(28B)–C(27B)	–179.7(6)
C(26B)–C(27B)–C(28B)–C(19B)	–1.0(10)

Symmetry transformations used to generate equivalent atoms:
